# Epigenomic reprogramming via HRP2-MINA dictates response to proteasome inhibitors in multiple myeloma with t(4;14) translocation

**DOI:** 10.1172/JCI149526

**Published:** 2022-02-15

**Authors:** Jingjing Wang, Xu Zhu, Lin Dang, Hongmei Jiang, Ying Xie, Xin Li, Jing Guo, Yixuan Wang, Ziyi Peng, Mengqi Wang, Jingya Wang, Sheng Wang, Qian Li, Yafei Wang, Qiang Wang, Lingqun Ye, Lirong Zhang, Zhiqiang Liu

**Affiliations:** 1 The Province and Ministry Co-sponsored Collaborative Innovation Center for Medical Epigenetics, Tianjin Key Laboratory of Cellular Homeostasis and Human Diseases, Department of Physiology and Pathophysiology, School of Basic Medical Science, Tianjin Medical University, Heping, Tianjin, China.; 2 2011 Collaborative Innovation Center of Tianjin for Medical Epigenetics, Tianjin Key Laboratory of Medical Epigenetics, Key Laboratory of Immune Microenvironment and Disease (Ministry of Education), Department of Biochemistry and Molecular Biology, School of Basic Medical Sciences, Tianjin Medical University, Tianjin, China.; 3 Tianjin Medical University Cancer Institute and Hospital, National Clinical Research Center for Cancer, Tianjin Key Laboratory of Cancer Prevention and Therapy, Tianjin Clinical Research Center for Cancer, Tianjin, China.; 4 Center for Translational Research in Hematological Malignancies, Cancer Center, Houston Methodist Hospital, Houston, Texas, USA.

**Keywords:** Cell Biology, Hematology, Apoptosis survival pathways, Drug therapy, Tumor suppressors

## Abstract

The chromosomal t(4;14) (p16;q32) translocation drives high expression of histone methyltransferase nuclear SET domain–containing 2 (NSD2) and plays vital roles in multiple myeloma (MM) evolution and progression. However, the mechanisms of NSD2-driven epigenomic alterations in chemoresistance to proteasome inhibitors (PIs) are not fully understood. Using a CRISPR/Cas9 sgRNA library in a bone marrow–bearing MM model, we found that hepatoma-derived growth factor 2 (HRP2) was a suppressor of chemoresistance to PIs and that its downregulation correlated with a poor response and worse outcomes in the clinic. We observed suppression of HRP2 in bortezomib-resistant MM cells, and knockdown of HRP2 induced a marked tolerance to PIs. Moreover, knockdown of HRP2 augmented H3K27me3 levels, consequentially intensifying transcriptome alterations promoting cell survival and restriction of ER stress. Mechanistically, HRP2 recognized H3K36me2 and recruited the histone demethylase MYC-induced nuclear antigen (MINA) to remove H3K27me3. Tazemetostat, a highly selective epigenetic inhibitor that reduces H3K27me3 levels, synergistically sensitized the anti-MM effects of bortezomib both in vitro and in vivo. Collectively, these results provide a better understanding of the origin of chemoresistance in patients with MM with the t(4;14) translocation and a rationale for managing patients with MM who have different genomic backgrounds.

## Graphical abstract

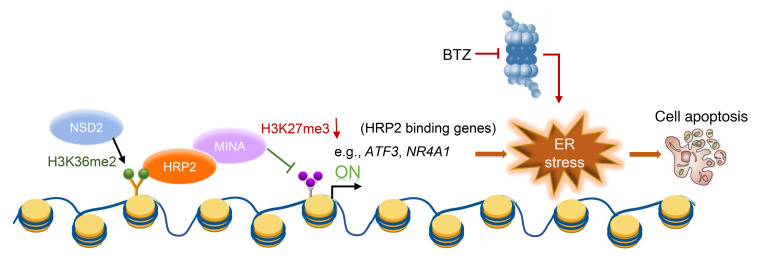

## Introduction

Multiple myeloma (MM) is a genetically complex and heterogeneous hematologic malignancy characterized by the monoclonal expansion of malignant plasma cells, leading to numerous genetic abnormalities including chromosomal translocations, deletions, duplications, and genetic mutations (1, 2). Clinically, translocations involving the IgH chain region at chromosome 14q32 occur in approximately 40% of patients with MM, which contributes to disease progression and therapeutic resistance (3). According to the updated diagnostic criteria, t(4;14) translocation is one of the high risks that confers a poor prognosis for patients with MM (4). Despite the fact that proteasome inhibitor–based (PI-based) regimens show remarkable benefits in the large majority of patients with newly diagnosed MM, low complete response (CR) rates and resistance to chemotherapies are still major challenges in the clinic. Therefore, understanding the mechanisms underlying the initiation of drug resistance in high-risk MM patients may improve their outcomes and pave the way for personalized medicine.

The t(4;14) (p16;q32) translocation confers high expression of fibroblast growth factor receptor 3 (FGFR3) and nuclear SET domain–containing 2 (NSD2, also known as WHSC1/MMSET) genes and is one of the most common translocations in patients with MM, accounting for 15% to 20% of all chromosomal abnormalities (5). NSD2 is a SET domain–containing histone methyltransferase (HMT) that specifically catalyzes H3K36 dimethylation (H3K36me2; ref. 6). NSD2 is involved in the proliferation, apoptosis, and adhesion of MM cells, and the HMT activity of NSD2 is critical for its biological function in tumorigenicity (7). Overexpression or gain-of-function mutation in NSD2 results in drug resistance in multiple cancers (8–10) and drives endocrine resistance via the reprogramming of metabolism by coordinating pentose phosphate pathway enzymes (11). Although a recent retrospective study showed that t(4;14) translocations are associated with high-risk disease characteristics in patients with MM, they are also associated with better responses to PI-based treatments (12). In fact, another study also concluded that patients with MM who were t(4;14) positive were chemotherapy sensitive but easily relapsed (13). Specifically, Shah et al. reported that NSD2^hi^ MM cells were resistant to melphalan treatment because of an enhanced DNA damage repair capacity (14). However, it remains unknown whether NSD2 plays critical roles in PI-induced drug resistance in MM cells and how it epigenetically drives chemosensitivity.

Histone H3K36 methylation is a well-known hallmark of active transcription (15). In patients with MM who have the t(4;14) translocation, overexpressed NSD2 causes global augmentation in H3K36me2, alters distribution across the genome, and leads to a concomitant genome-wide reduction of H3K27 methylation (7, 16, 17). Biologically, histone lysine methylation serves as a scaffold for reader modules, and the latter exert their functions by recognizing the methylated site via methyl-lysine binding motifs (18, 19). HRP2 (also known as HDGFRP2) is a structurally related member of the family of hepatoma-derived growth factor–related proteins, all of which are characterized by a conserved HATH/PWWP domain at the N-terminus (20). HRP2 preferentially binds to histone peptides characteristic of transcriptionally inactive chromatin and is a reader of H3K36me3/2 (21). HRP2 plays a role in the integration of HIV-1–infected cells (22), forms a complex with DPF3a and BAF in activating gene transcription during myogenic differentiation (23), and promotes DNA repair by homologous recombination (21). In cancer, HRP2 promotes cell growth in hepatocellular carcinoma (HCC), mainly by interacting with the RNA processing regulator IWS1, and positively regulates mRNA levels of cyclin D1 (24). However, the expression, biological functions, and regulatory mechanism of HRP2 in hematologic malignancies, especially in MM, are unknown.

In this study, we applied a CRISPR/Cas9 sgRNA screen system and identified genes responsible for sensitivity to bortezomib in a bone marrow–bearing MM growth model of NOD/SCID (NSG) mice and identified HRP2 as a suppressor of chemoresistance to PIs. We explored the mechanism by which HRP2 mediates chromatin modifications and transcriptome alterations, making cells prone to chemosensitivity and apoptosis, and evaluated the translational significance in combination with PIs both in vitro and in vivo.

## Results

### CRISPR library screening identifies HRP2 as a critical gene for sensitivity of MM to bortezomib treatment.

**Figure 1:**
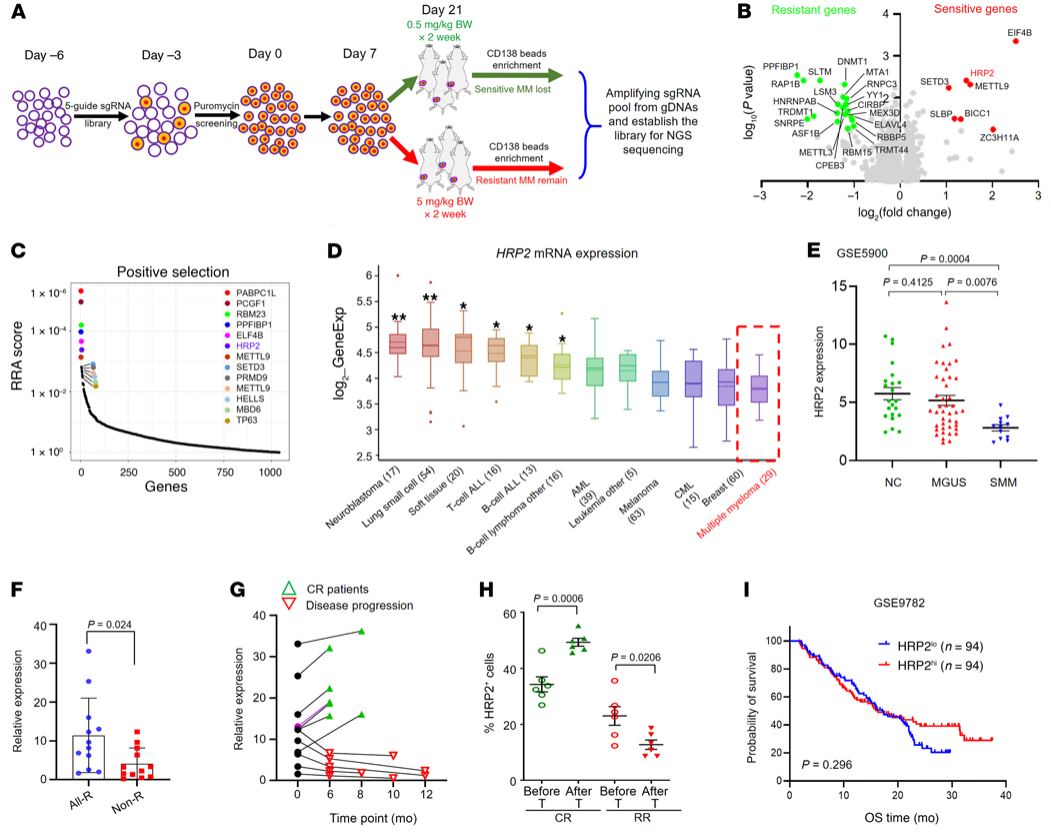
In vivo CRISPR library screening identified HRP2 as a key negative regulator of bortezomib resistance. (**A**) Diagram of the genome-wide CRISPR/Cas9 screening system in a NSG femur bone marrow–bearing MM model. The 3 most obviously changed tumors from 9 mice per group were chosen for screening. gDNA, genomic DNA. (**B**) Volcano plot illustrating the depleted genes in the negative selection and the enriched genes in the positive selection. (**C**) Illustration of the top 10 gene candidates from the above screening. (**D**) HRP2 expression in 12 categories of tumor samples from the CCLE database. *P < 0.05 and **P < 0.02, by 2-sided Student’s *t* test. (**E**) Expression of HRP2 in bone marrow plasma cells from healthy donors (nontarget control [NT ctrl], *n* = 22), patients with MGUS (*n* = 44), and patients with SMM (*n* = 12) from the GSE5900 cohort. ALL, acute lymphocytic leukemia; AML, acute myeloid leukemia; CML, chronic myelogenous leukemia. (**F**) *HRP2* expression in patients with MM showed all kinds of responses (All-R, *n =* 12) or a nonresponse (Non-R, *n =* 12). (**G**) Expression trend of the *HRP2* gene before and after a bortezomib-based treatment regimen: 7 patients with MM showed a CR and 5 patients with MM showed disease progression. (**H**) Quantification of HRP2^+^ cells in an immunohistochemical assay for 6 patients with a CR and 6 patients with RR bone marrow biopsies. T, treatment. (**I**) Correlation of *HRP2* mRNA expression with overall survival (OS) in myeloma patients from Mulligan’s database (*n =* 188, GSID: GS-DT-52). The cutoff was the median of HRP2 expression. *P* values were determined by Pearson’s coefficient and log-rank test (**I**) and Student’s *t* test (**E**, **F**, and **H**).

To identify the critical genes governing sensitivity of MM cells to PIs, we performed genome-wide CRISPR/Cas9-KO screening using a library of 70,290 sgRNAs targeting all human genes (25). Luciferase-labeled LP-1 cells were infected with a lentivirus-carrying library with a MOI of 30% or less, and positively infected cells were selected by puromycin and expanded without significant loss of sgRNAs. To maximally mimic the bone marrow microenvironment, we injected LP-1 cells into the femur bone marrow of NSG mice and evaluated tumor growth by monitoring luciferase activity. Then we treated the mice with 0.5 or 5 mg/kg bortezomib and found that 0.5 mg/kg had no significant anti-MM effect, whereas 5 mg/kg killed almost all MM cells (Supplemental Figure 1A; supplemental material available online with this article; https://doi.org/10.1172/JCI149526DS1). Thus, we screened genes for bortezomib resistance by negative selection and bortezomib-sensitive genes by positive selection (Figure 1A). The coverage of sgRNAs in the cells was calculated by high-throughput sequencing after amplification of the sgRNA sequence in the genome; we found 28 genes through positive selection in the sensitive cells and 15 genes through negative selection in the resistant cells (Figure 1B). We validated the expression of the top 10 genes from the negative and positive selections in our previously established bortezomib-resistant MM cells and identified *HRP2* as the most differentially expressed one among the enriched genes (Supplemental Figure 1B). All *HRP2*-targeting sgRNAs were markedly increased in bortezomib-resistant cells, and the robust rank aggregation (RRA) algorithm confirmed that *HRP2* was one of the most essential genes (Figure 1C), indicating that loss of *HRP2* might desensitize MM cells to bortezomib treatment. In addition, when using the Broad Institute’s Cancer Cell Line Encyclopedia (CCLE), we found that *HRP2* expression was downregulated in tumor cells of hematopoietic and lymphoid tissues compared with expression in normal tissues (Supplemental Figure 1C), and was the lowest in patients with MM among all hematological cancers (Figure 1D). Clinically, a gene array assay using plasma cells from healthy donors, patients with monoclonal gammopathy of undetermined significance (MGUS), and patients with smoldering MM (SMM) revealed no obvious difference in HRP2 expression between healthy donors and patients with MGUS, but significantly lower expression was detected in plasma cells of patients whose disease had progressed to SMM (Figure 1E). Additionally, in Carrasco’s MM cohort from the Oncomine database, HRP2 expression was suppressed in patients with MM, with recurrence after 1 year of treatment (Supplemental Figure 1D). In our cohort, *HRP2* expression was significantly lower in nonresponders to bortezomib-based treatment compared with expression in responders (Figure 1F), and downregulated HRP2 predicted refractory or relapsed (RR) disease progression, but elevated *HRP2* predicted a CR during treatment (Figure 1G). Correspondingly, HRP2 protein levels were also clearly increased in biopsies from patients with MM who had a CR but were suppressed in those with disease progression (Figure 1H). An independent cohort of patients with relapsed MM after single bortezomib treatment (APEX, SUMMIT, and CREST trials; *n =* 188) from Mulligan’s study (26) indicated that low expression of HRP2 predicted a worse trend of overall survival (OS) (Figure 1I) that was also seen in Zhan’s cohort of pretreatment bone marrow aspirates from patients with MM (Supplemental Figure 1E and ref. 27). These data suggest that HRP2 is closely correlated with the treatment response and outcomes of patients with MM in the clinic.

### Depletion of HRP2 induces chemoresistance to PIs in vitro and in vivo.

**Figure 2:**
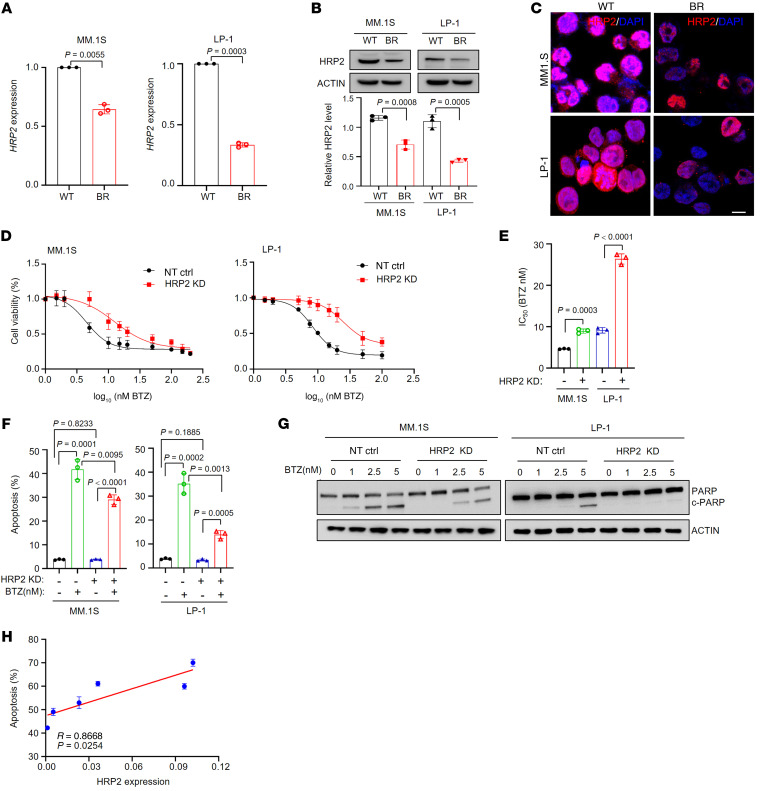
KD of HRP2 induces resistance to PIs in MM cells. HRP2 (**A**) mRNA and (**B**) protein expression and quantification in WT and bortezomib-resistant (BR) MM.1S and LP-1 cells (*n =* 3). Two-sided *P* values in **A** and **B** were determined by Student’s *t* test. Data indicate the mean ± SD. (**C**) Immunofluorescence analysis for HRP2 (red) in WT and bortezomib-resistant MM.1S and LP-1 cells. Nuclei were stained with DAPI (blue). Scale bar: 10 μm. *n =* 3. (**D**) Alteration of the IC_50_ to bortezomib (BTZ) in the nontarget control (NT ctrl) and HRP2-KD MM.1S and LP-1 cells. (**E**) Comparison of the IC_50_of bortezomib in nontarget control and HRP2-KD MM.1S and LP-1 cells (*n =* 3). Two-sided *P* values were determined by Student’s *t* test. Data indicate the mean ± SD. (**F**) Flow cytometric analysis of apoptosis of the nontarget control and HRP2-KD MM.1S and LP-1 cells, respectively, following bortezomib treatment for 48 hours (*n =* 3). Two-sided *P* values were determined by Student’s *t* test. Data indicate the mean ± SD. (**G**) Cleavage of PARP (c-PARP) in the nontarget control and HRP2-KD MM.1S and LP-1 cells treated with increasing dosages of bortezomib for 48 hours (*n =* 3). (**H**) Correlation coefficient between HRP2 expression and apoptosis rates following bortezomib treatment in samples from patients with MM (*n =* 6). Two-sided *P* values were determined by Pearson’s coefficient and log-rank tests. Data indicate the mean ± SD of 3 independent experiments.

As low HRP2 expression is associated with poor outcomes in patients with MM, we evaluated HRP2 expression in 2 of our previously established bortezomib-resistant MM cell lines derived from MM.1S and LP-1 cells (28). HRP2 mRNA and protein expression levels were significantly suppressed, but the changes were greater in LP-1 cells than in MM.1S cells (Figure 2, A and B), and immunofluorescence staining revealed suppressed expression of HRP2 in bortezomib-resistant MM cells (Figure 2C). Next, we evaluated the effect of HRP2 depletion on the proliferation and apoptosis of MM.1S and LP-1 cells. After HRP2 was successfully deleted by a sgRNA (Supplemental Figure 2A), we found that cell proliferation was not meaningfully affected (Supplemental Figure 2B); however, both MM.1S and LP-1 cells became resistant to bortezomib (Figure 2D), as evidenced by the elevated IC_50_ values (Figure 2E). Meanwhile, sensitivity to another PI, carfilzomib, was also substantially inhibited in MM cells with HRP2 depletion (Supplemental Figure 2, C–E), but no considerable alterations were observed in response to melphalan or dexamethasone (Supplemental Figure 2, F, and G). Variation in sensitivity to PIs was also confirmed by the significant decline in apoptosis rates induced by bortezomib (Figure 2F) and the decrease in cleaved poly(ADP-ribose)polymerase (PARP) (Figure 2, G and H). Intriguingly, we found a significant positive correlation between the apoptosis rate and HRP2 levels in six CD138^+^ plasma cells from t(4;14)^+^ patients with MM (Figure 2H). On the other hand, when HRP2 was overexpressed (Supplemental Figure 3A), MM cells became more sensitive to PI treatment (Supplemental Figure 3, B and C) and more easily induced apoptosis (Supplemental Figure 3, D–F). Collectively, these data strongly indicate that HRP2 is a key regulator of sensitivity to PIs in MM cells.

**Figure 3:**
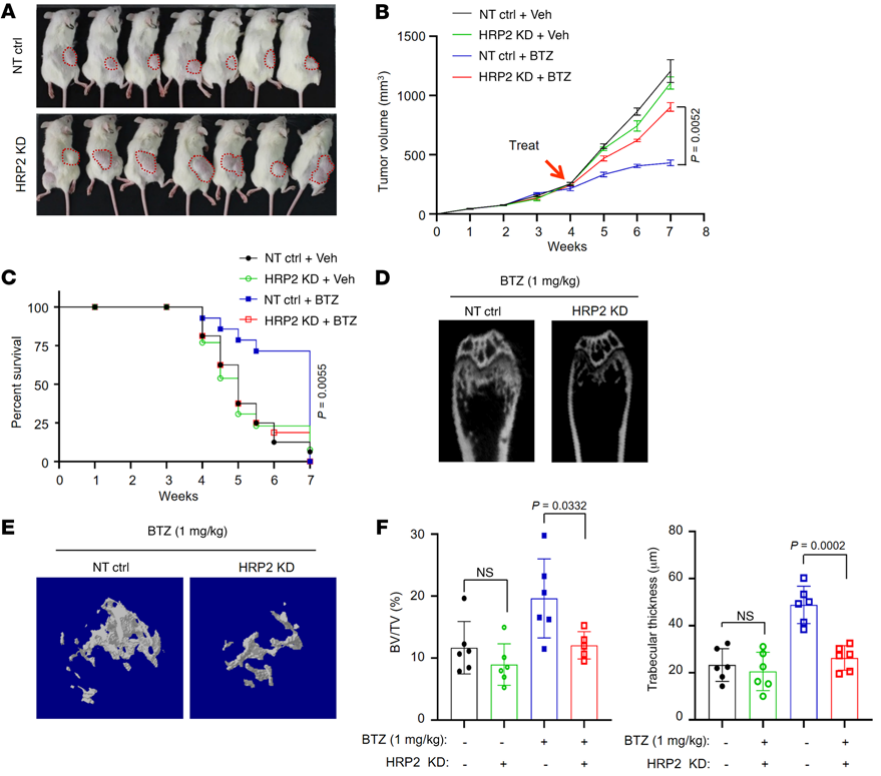
Depletion of HRP2 enhances bortezomib resistance in vivo. (**A**) Images of xenografts derived from LP-1 nontarget control (NT ctrl) and HRP2-KD cells (2 × 10^6^ cells/mouse, *n =* 7 mice/group) in NSG mice after the end of bortezomib treatment. (**B**) Growth curve of tumors in mice that received DMSO (Veh) or bortezomib (1 mg/kg) and bore tumors derived from LP-1 nontarget control or HRP2-KD cells (*n =* 7 mice/group). Arrows indicate the point at which treatment began (day 25). Differences between groups were assessed by 1-way ANOVA. Data indicate the mean ± SD. (**C**) Kaplan-Meier curves showing survival of mice treated with bortezomib or vehicle control (*n =* 7 mice/group). Two-sided *P* value was determined by log-rank test. Data indicate the mean ± SD. (**D**) Representative micro-CT reconstructions of mouse femurs bearing nontarget control or HRP2-KD LP-1 cells (5 × 10^5^ cells/mouse) and treated with bortezomib (1 mg/kg; *n =* 6 mice/group) and (**E**) 3D reconstructions of bone trabecula in metaphyseal regions (*n =* 6 mice/group). (**F**) Ratio of bone volume to total volume (BV/TV) and trabecular thickness in the metaphyseal regions of mouse femurs (*n =* 6 mice/group). Two-sided *P* values were determined by Student’s *t* test. Data indicate the mean ± SD.

When we evaluated the in vivo effects of HRP2 depletion on resistance to bortezomib treatment, we found that HRP2 depletion did not affect tumor growth in a xenograft model, but rather induced tolerance to bortezomib treatment compared with the nontarget controls (Figure 3, A and B) and significantly impaired the survival rate of mice (Figure 3C). The effect of HRP2 depletion on bortezomib resistance was also reflected by the deteriorated bone lesion when these cells were inoculated into the bone marrow of NSG mice that underwent bortezomib treatment (Figure 3, D and E) and by the restrained restoration of the bone structure (Figure 3F). These data confirm the important role of HRP2 in inducing bortezomib resistance in MM cells.

### HRP2 depletion mediates transcriptome alterations that facilitate MM cell survival.

**Figure 4:**
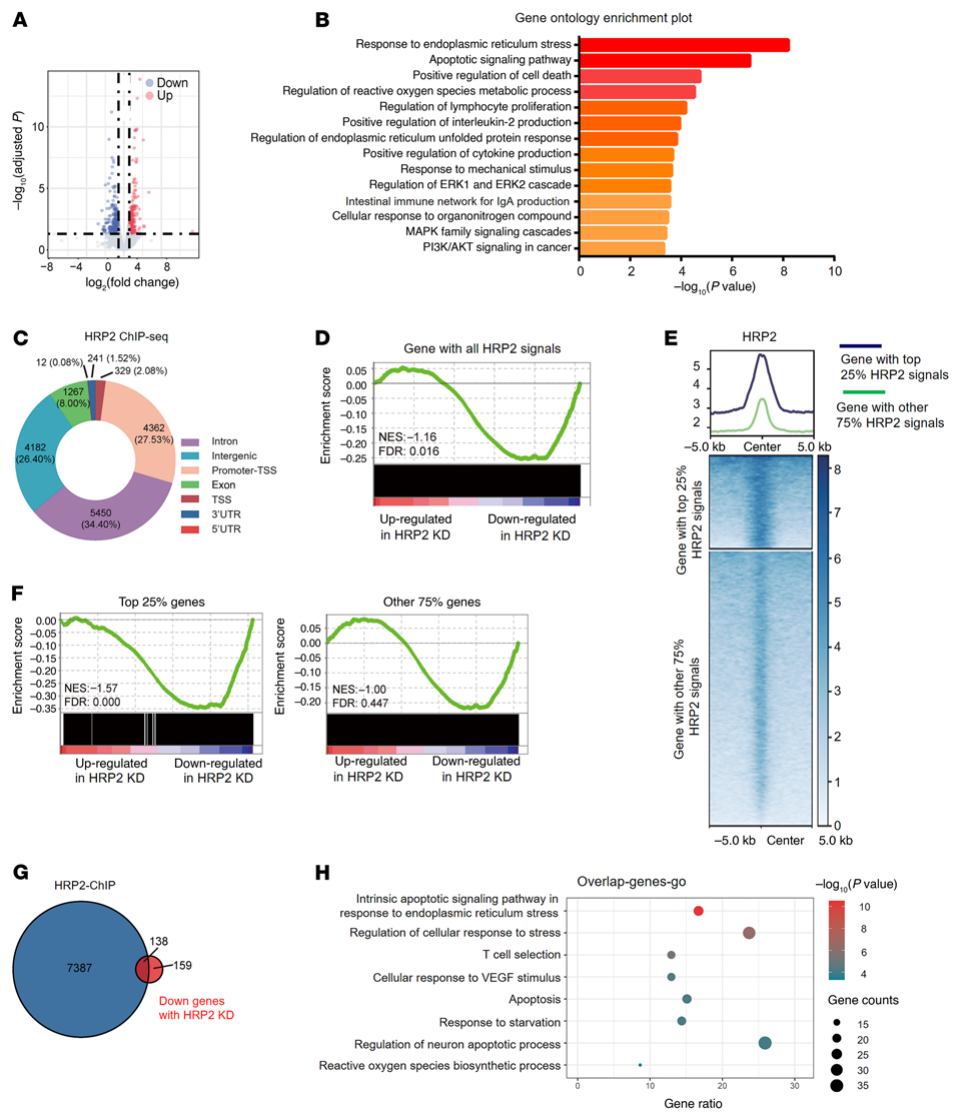
Transcriptomic analysis reveals HRP2 to be a negative regulator of ER stress. (**A**) Volcano plot of DEGs analyzed from bulk RNA-Seq of LP-1 HRP2-KD cells compared with the nontarget control. Downregulated genes (Down) are shown in blue; upregulated genes (Up) are shown in red; and statistically nonsignificant genes are shown in light blue (*n =* 2 independent biological experiments). (**B**) GO analysis of DEGs with a *P* value of less than 0.05 using DAVID (Database for Annotation, Visualization, and Integrated Discovery) methods. (**C**) Genome-wide distribution of HRP2 binding regions in LP-1 cells. (**D**) GSEA enrichment plot of HRP2 binding genes and RNA-Seq analysis. NES, normalized enrichment score. (**E**) HRP2 binding genes were categorized into 2 subgroups according to HRP2 signals on these genes. (**F**) GSEA of the top 25% of genes with HRP2 signals and the other 75% of genes with HRP2 signals, shown for comparison. (**G**) Venn diagram showing the number of overlaps between genes bound by HRP2 and downregulated genes upon HRP2 depletion. (**H**) GO enrichment analysis of overlapping genes in combined RNA-Seq and ChIP-Seq analyses.

We further explored the regulatory mechanism of HRP2 in dictating sensitivity to bortezomib treatment in MM cells. Since HRP2 has been implicated in transcriptional regulation in myogenesis (23, 29), we performed RNA-Seq analysis and identified differentially expressed genes (DEGs) in LP-1 cells upon HRP2 depletion. We identified 280 upregulated and 297 downregulated genes in the HRP2-depleted cells (Figure 4A). Gene Ontology (GO) analysis indicated that downregulated genes were significantly enriched for the regulation of cell apoptosis and cell death (Figure 4B), consistent with the role of HRP2 in enhancing cell susceptibility to drug-induced apoptosis in MM cells. To further interpret HRP2’s function in regulating genes responsible for the apoptosis of MM cells, we mapped its genome-wide distribution by ChIP-Seq in LP-1 cells and found a large proportion of HRP2 distributed on the gene promoter and transcription start site (TSS) regions (Figure 4C). Integrated with RNA-Seq data, gene set enrichment analysis (GSEA) showed that HRP2-bound genes were significantly enriched in downregulated genes upon HRP2 depletion (Figure 4D). Furthermore, the enrichment scores were positively correlated with HRP2 occupancies on its target genes, especially when we compared the top 25% of genes with HRP2 signals (Figure 4, E and F). Combining these 2 data sets, we found that nearly 50% of downregulated genes overlapped with HRP2-bound genes (Figure 4G), and these genes were substantially enriched for apoptosis in response to ER stress (Figure 4H). These results suggest that loss of HRP2 desensitized MM cells to bortezomib treatment by regulating genes involved in apoptotic pathways in response to ER stress. Indeed, evaluated the phosphorylation status of JNK, a key regulator of ER stress, in LP-1 cells upon bortezomib treatment, but observed no appreciable change in the HRP2-depleted cells (Supplemental Figure 4A). The change in expression of the ER stress downstream target gene *CHOP* was similar (Supplemental Figure 4, B and C). Suppression of phosphorylated JNK (p-JNK) and ER stress target genes was much weaker in MM.1S cells than in LP-1 cells (Supplemental Figure 4, D–F).

### Functions of HRP2 are NSD2 dependent.

**Figure 5:**
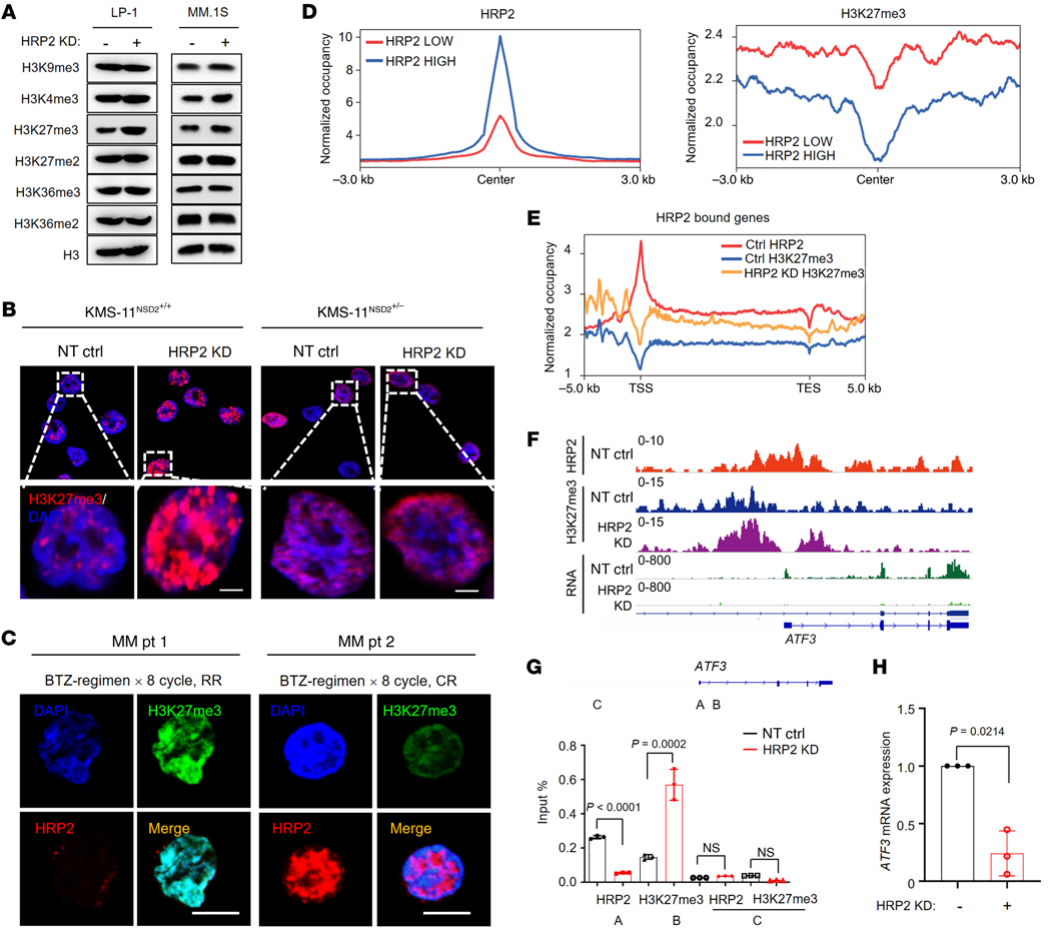
Silencing of HRP2 upregulates H3K27me3 modification. (**A**) Western blotting detecting the 6 most common histone methylation modifications in LP-1 and MM.1S cells infected with lentivirus carrying a nontarget control or an shRNA targeting HRP2 (HRP2 KD). *n =* 3. (**B**) Confocal fluorescence images showing H3K27me3 expression levels in KMS11^NSD2+/+^ and KMS11^NSD2+/–^ cells with or without HRP2 KD. Scale bars: 10 μm. Original magnification, ×100 (top panels). *n =* 3. (**C**) Confocal fluorescence images of HRP2 (red) and H3K27me3 (green) in CD138^+^ plasma cells from patients with MM (patients with a CR, *n =* 3; patients with RR, *n =* 3). Scale bars: 10 μm. (**D**) Tag density profile of HRP2 and H3K27me3 distribution in LP-1 cells. (**E**) Tag density profile of HRP2 and H3K27me3 distribution on HRP2-bound genes in LP-1 cells. TES, transcription end site. (**F**) Gene tracks showing representative ChIP-Seq profiles for the indicated proteins and histone marks at the *ATF3* gene loci. (**G**) ChIP-qPCR of H3K27me3 and HRP2 at the *ATF3* gene loci in HRP2-KD LP-1 cells (*n =* 3). PCR primers were designed according to ChIP-Seq peaks of the corresponding proteins on these gene loci. A schematic representation of the PCR primer design is shown. A, TSS; B, coding region; C, intergenic region. Two-sided *P* values were determined by Student’s *t* test. Data indicate the mean ± SD. (**H**) qPCR analyses of *ATF3* mRNA expression levels in HRP2-KD LP-1 cells compared with the nontarget control (*n =* 3). Two-sided *P* value was determined by Student’s *t* test. Data indicate the mean ± SD.

Next, we determined how HRP2 participates in the transcription of genes prone to MM cell apoptosis through epigenetic regulation. We examined the 6 most common histone methylation marks in LP-1 and MM.1S cells and found that H3K4me3 expression slightly increased in both cells, but H3K27me3 expression was substantially increased upon HRP2 depletion only in LP-1 cells (Figure 5A and Supplemental Figure 5A). We consistently observed obviously enhanced H3K27me3 foci in the KMS-11^NSD2+/+^ cell nucleus upon HRP2 depletion but, intriguingly, not in KMS-11^NSD2+/–^ cells (Figure 5B). Importantly, we also found a negative correlation between HRP2 and H3K27me3 levels in CD138^+^ cells isolated from t(4;14)-positive patients with MM, in which patients with a CR after 8 cycles of a bortezomib-based treatment regimen had higher HRP2 expression and lower H3K27me3 expression, and vice versa for patients with RR (Figure 5C and Supplemental Figure 5B). These data indicate that HRP2 may regulate H3K27me3 levels, and this regulatory machinery may depend on NSD2 abundance or rather the corresponding histone mark H3K36me2 (23). To gain insights into whether HRP2 abundance negatively correlates with H3K27me3 on its target genes, we performed ChIP-Seq in LP-1 cells using H3K27me3 antibody. We categorized HRP2-bound genes into 2 groups according to HRP2-normalized tag density and found that genes with higher HRP2 enrichment had lower enrichment of H3K27me3 (Figure 5D). Moreover, metagene analysis and representative gene tracks showed that the normalized tag density of H3K27me3 was markedly increased around the TSS of HRP2-bound genes upon HRP2 depletion (Figure 5, E and F). Notably, H3K27me3 expression on some essential genes involved in ER stress-related apoptotic pathways was elevated upon HRP2 knockdown (Supplemental Figure 5C). These essential genes included the activating transcription factor 3 (*ATF3*) (ref. 30 and Figure 5, G and H) and the tumor suppressor gene nerve growth factor 1B (*NR4A1*) (ref. 31, Supplemental Figure 5D, and Supplemental Figure 6A), suggesting that they were tightly regulated by HRP2 and H3K27me3. In addition, expression of these target genes decreased meaningfully in the bortezomib-resistant and HRP2-knockdown (HRP2-KD) MM cells compared with their control group cells (Supplemental Figure 6, B and C). Functionally, overexpression of *ATF3* and *NR4A1* partially attenuated the HRP2-KD–induced resistance to PIs, both through enhanced ER stress and augmented apoptosis in the LP-1 cells with HRP2 KD (Supplemental Figure 6, D–F). Collectively, these findings indicate that HRP2 negatively regulated H3K27me3 levels and the transcription of genes governing bortezomib resistance in MM cells.

### HRP2 recruits MYC-induced nuclear antigen to demethylate H3K27me3 in MM cells.

**Figure 6:**
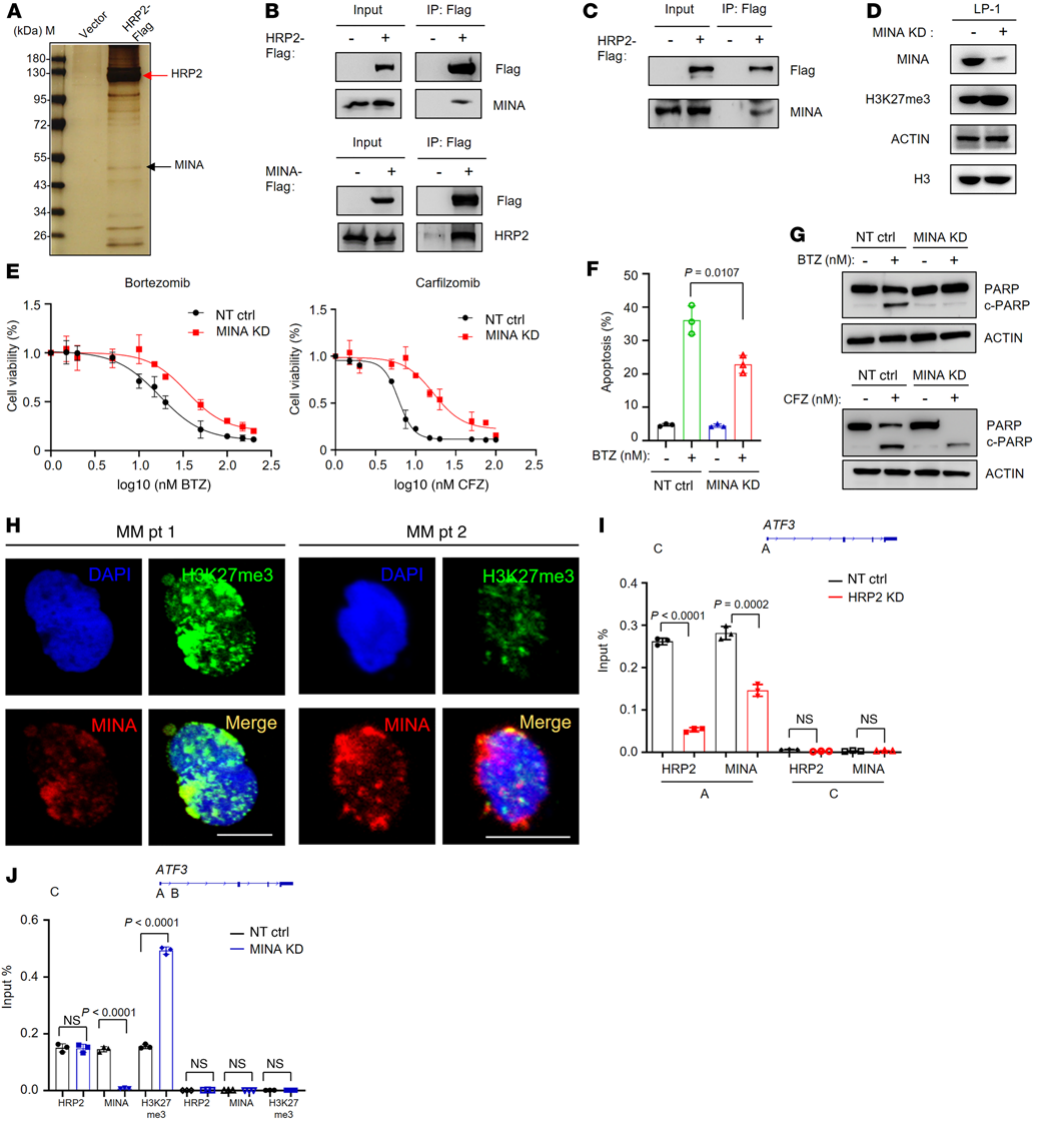
HRP2 recruits MINA to demethylate H3K27me3. (**A**) Total extracts of cells transiently expressing empty vector and HRP2-Flag (3×) were immunoprecipitated with anti-Flag M2-coupled agarose beads. Isolated HRP2 proteins that bound with silver prior to mass spectrometric analysis. (**B**) Co-IP assay of Flag-HRP2 and Flag-MINA in HEK293T cells (*n =* 3). (**C**) Co-IP analysis of the interactions between HRP2 and MINA complex subunits in LP-1 cells (*n =* 3). (**D**) MINA and H3K27me3 levels in LP-1 cells with MINA KD by a lentivirus-carrying shRNA (*n =* 3). (**E**) Alteration of cell viability following exposure to bortezomib in nontarget control and MINA-KD LP-1 cells (*n =* 3). (**F**) Flow cytometric analysis of apoptosis in nontarget control and MINA-KD LP-1 cells treated with 5 nM bortezomib for 48 hours (*n =* 3). Two-sided *P* value was determined by Student’s *t* test. Data indicate the mean ± SD. (**G**) Cleavage of PARP in nontarget control and MINA-KD LP-1 cells treated with bortezomib (5 nM) for 48 hours (*n =* 3). CFZ, carfilzomib. (**H**) Confocal fluorescence images of H3K27me3 and MINA levels in CD138^+^ plasma cells from patients with MM (*n =* 2). Scale bars: 10 μm. (**I**) ChIP-qPCR of HRP2 and MINA at the *ATF3* gene loci in HRP2-KD LP-1 cells (*n =* 3). PCR primers were designed according to ChIP-Seq peaks of the corresponding proteins on these gene loci. A schematic representation of PCR primer design is shown. Two-sided *P* values were determined by Student’s *t* test. Data indicate the mean ± SD. (**J**) ChIP-qPCR of HRP2, MINA, and H3K27me3 at the *ATF3* gene loci in MINA-KD LP-1 cells (*n =* 3). PCR primers were designed according to ChIP-Seq peaks of the corresponding proteins on these gene loci. Two-sided *P* values were determined by Student’s *t* test. Data indicate the mean ± SD.

To further investigate how HRP2 negatively regulates the H3K27me3 histone mark, we identified its binding partners that may participate in this process. We performed HRP2 co-IP using Flag antibody with extracts from Flag-HRP2–expressing cells. Mass spectrometric analysis identified a histone lysine demethylase, MYC-induced nuclear antigen (MINA, also known as MINA53), which is involved in the demethylation of H3K9me3 and H3K27me3 (32) and is enriched in the HRP2 complex (Figure 6A). We confirmed the association between HRP2 and MINA exogenously in HEK293T cells and MM cells by a co-IP assay (Figure 6, B and C). These results demonstrate that HRP2 associated with MINA. When MINA expression was successfully knocked down using a lentivirus harboring shRNA in LP-1 cells (Supplemental Figure 7A), H3K27me3 levels were substantially increased upon MINA depletion (Supplemental Figure 7, B and C and Figure 6D). At the same time, MINA depletion conferred LP-1 cells with a greater resistance to bortezomib- and carfilzomib-induced cell death and apoptosis (Figure 6, E and F) and suppressed the cleavage of PARP induced by bortezomib (Figure 6G and Supplemental Figure 7D). Clinically, in CD138^+^ plasma cells isolated from t(4;14)-positive patients with MM, protein levels of MINA and H3K27me3 were found to be repellent (Figure 6H). In addition, ChIP–quantitative PCR (ChIP-qPCR) analysis of the above-identified HRP2-bound genes, *ATF3* and *NR4A1*, revealed that depletion of HRP2 specifically decreased MINA signals on HRP2 binding sites (Figure 6I and Supplemental Figure 7E). MINA depletion also increased H3K27me3 signals around these regions (Figure 6J and Supplemental Figure 7F). Collectively, these data demonstrate that HRP2 was required for the recruitment of MINA to the TSS of target genes and could remove the H3K27me3 histone mark at these regions.

### Targeting H3K27me3 sensitizes bortezomib treatment in HRP2^lo^ MM cells in vitro and in vivo.

**Figure 7:**
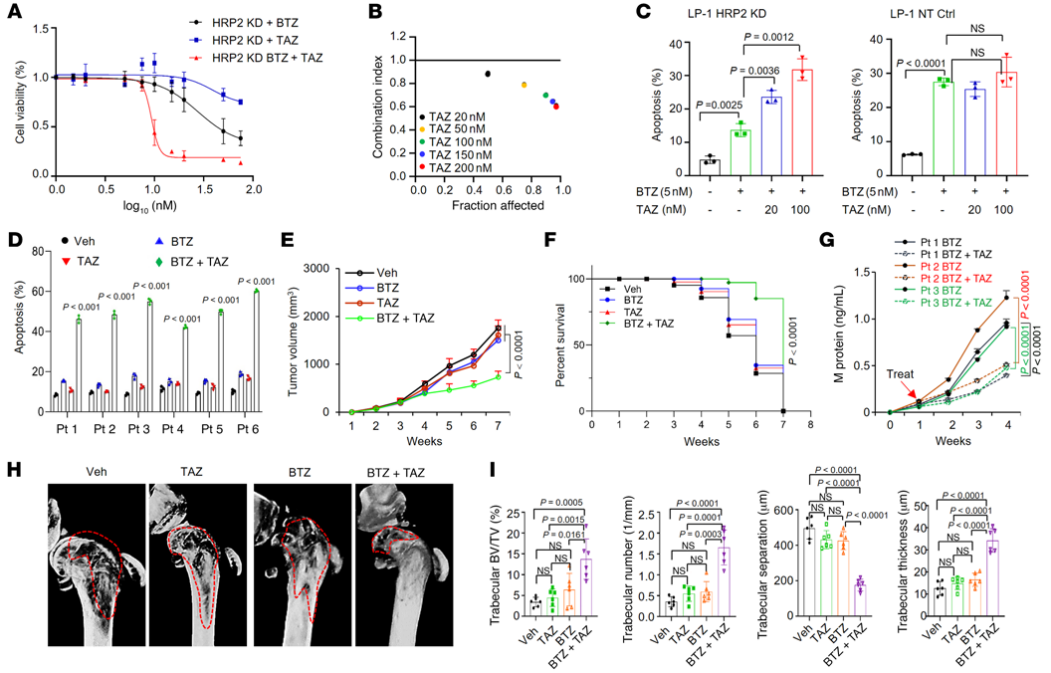
Tazemetostat sensitizes HRP2^lo^ MM cells to bortezomib treatment in vitro and in vivo. (**A**) Cell viability of HRP2-KD LP-1 cells treated with 5 nM bortezomib and various concentrations of tazemetostat (TAZ) for 24 hours (*n =* 3). Data indicate the mean ± SD. (**B**) CI plots of tazemetostat and bortezomib combinations in HRP2-KD LP-1 cells. A CI of less than 1 defines a synergistic effect of 2 reagents (*n =* 3). (**C**) Apoptosis of nontarget control and HRP2-KD cells treated with bortezomib (5 nM) in the presence or absence of 2 dosages of tazemetostat for 48 hours (*n =* 3). Two-sided *P* values were determined by Student’s *t* test. Data indicate the mean ± SD. (**D**) Apoptosis of CD138^+^ plasma cells from patients with t(4;14) RR MM (*n =* 6). *n =* 3. Two-sided *P* values were determined by Student’s *t* test. Data indicate the mean ± SD. (**E**) Tumor growth of bortezomib-resistant LP-1 cells (3 × 10^6^ cells/mouse) in NSG mice treated with vehicle, bortezomib, tazemetostat, or bortezomib plus tazemetostat (bortezomib, 1 mg/kg, i.p.; tazemetostat, 0.5 mg/kg, po; *n =* 12 mice/group). Two-sided *P* value of the mean ± SD was determined by 2-way ANOVA. (**F**) Survival rates of mice when their tumors had reached 15 mm^3^ in size (*n =* 12 mice/group). Two-sided *P* value was analyzed by log-rank test. Data indicate the mean ± SD. (**G**) Levels of M protein secreted in PDX mouse tail vein blood after treatment with bortezomib or with bortezomib plus tazemetostat (bortezomib, 1 mg/kg, i.p.; tazemetostat, 0.5 mg/kg, po; *n =* 6 mice/patient, *n =* 3 mice/group). *P* values were determined by Pearson’s coefficient and log-rank tests. (**H**) Representative micro-CT reconstructions of femurs bearing bortezomib-resistant LP-1 cells (5 × 10^5^ cells/mouse; *n =* 8 mice/group). (**I**) Quantification of bone structure of mouse femurs (*n =* 6 mice/group). Two-sided *P* values of the mean ± SD were determined by Student’s *t* test.

To clarify the translational significance of our findings, we determined whether a synergistic anti-MM effect could be elicited when small molecules targeting H3K27me3 were combined with bortezomib (31). In HRP2-depleted LP-1 cells that were bortezomib resistant, administration of an inhibitor of tazemetostat, an inhibitor of the H3K27me3-catalyzing enzyme EZH2, failed to kill MM cells even at a very high dosage; however, it markedly improved the anti-MM effects of bortezomib (Figure 7A), and the combination effect was synergistic (Figure 7B). Moreover, this synergistic effect with tazemetostat only occurred in bortezomib-resistant and HRP2-depleted cells but not in parental or control LP-1 cells (Supplemental Figure 8A and Figure 7C), or in MM.1S cells without t(4;14) translocation (Supplemental Figure 8B). We also observed this phenotype with another EZH2 inhibitor, CPI169, in LP-1 cells with or without HRP2 depletion (Supplemental Figure 8C and D), as evidenced by the cleavage of PARP as a marker of apoptosis (Supplemental Figure 8E). Importantly, in CD138^+^ plasma cells isolated from six t(4;14)-positive patients with disease progression after treatment with bortezomib-based regimens, we found that a remarkable anti-MM effect exceeding the combination range was elicited (Figure 7D). Mechanistically, we found that tazemetostat induced *ATF3* and *NR4A1* expression, accompanied by decreased H3K27me3 levels on the promoters of these genes (Supplemental Figure 9, A–C).

The synergistic anti-MM effect of targeting H3K27me3 with PIs was evaluated in our previously established xenograft and bone destruction models. Using a bortezomib-resistant LP-1 cell–derived xenograft (CDX) model, we assessed the combination of tazemetostat and bortezomib in overcoming bortezomib resistance. Routine bortezomib (1 mg/kg) treatment alone failed to suppress bortezomib-resistant LP-1–derived tumor growth, but in the presence of tazemetostat (0.5 mg/kg), we observed successful rescue of the anti-MM effects of bortezomib to a large degree (Figure 7E) and significantly prolonged survival of the mice bearing these tumors compared with those treated with vehicle control or tazemetostat alone (Figure 7F). At the same time, expression of the downstream target genes *ATF3* and *NR4A1* was substantially increased in the combination treatment group compared with mice in the groups treated with vehicle control, bortezomib alone, or tazemetostat alone (Supplemental Figure 9D). Using 3 relapsed MM patients’ unsorted bone marrow mononuclear cells after bortezomib-based treatment, we established a patient-derived xenograft (PDX) model to assess the effects of the combination treatment on bortezomib-resistant MM cells. As expected, tumor burdens were all extenuated in the combination treatment groups, as reflected by significantly suppressed M protein levels (Figure 7G), as well as remarkably fewer CD138^+^ cells remaining in the murine bone marrow (Supplemental Figure 9E). Moreover, in the bortezomib-resistant LP-1 cell–derived bone destruction model, the combination of tazemetostat and bortezomib significantly alleviated bone disruption compared with mice in the vehicle control, bortezomib-alone, or tazemetostat-alone groups, as evidenced by shrinkage of the osteolytic lesion area and fewer cortical perforations following treatment (Figure 7H), in addition to a preserved trabecular network in the metaphyseal and diaphyseal regions of mouse femurs (Figure 7I and Supplemental 9F). Taken together, these data strongly suggest that targeting H3K27me3 in HRP2^lo^-expressing MM cells that are resistant to PIs resensitizes the anti-MM effects of PIs in vivo.

## Discussion

A low CR to PI-based regimens is still the main obstacle to managing high-risk patients with MM in the clinic, and epigenetic regulations are believed to play key roles in intrinsic or acquired chemoresistance. In this study, using an in vivo CRISPR/Cas9 screening system, we identified an unexpected role of an H3K36me2 reader protein, HRP2, in regulating PI sensitivity in MM cells in vitro, in vivo, and in the clinic. Our study further showed that HRP2 exerts its functions by reprogramming the transcriptional program that induces chemosensitivity in MM. Translationally, the small-molecule inhibitor tazemetostat synergistically enhanced sensitivity to bortezomib in HRP2^lo^ MM cells. Thus, our study provides new insights into the characteristics of treatment responses in high-risk patients with MM and sheds light on developing personalized strategies to manage patients with MM who have different cytogenetic backgrounds.

The CRISPR/Cas9-KO library screening system that has been created is a powerful tool for identifying genes that regulate chemosensitivity on the genome-wide or gene-set–focused scale (33–35). In this study, we adopted the GeCKO v2A library system integrating guild sgRNA and Cas9 together as previously described (36). We used a so-called positive and negative selection strategy, in which negative selection identified resistance-related genes, and positive selection identified those genes related to sensitivity. We also excluded the possibility that depletion of those genes yields self-apoptosis in MM cells via in vitro validation, because we found no obvious difference in the number of sgRNAs before and after propagation. Meanwhile, our intra-bone MM growth model could reflect the response to drug more intuitively through the monitoring of luciferase intensity. Thus, our study establishes a reliable in vivo screening system for selecting drug-responsive genes.

Using this unique selection system, we identified 28 genes in the negative selection group and 15 genes in the positive selection group. Among the genes identified in the screen, HRP2 promoted cell growth by affecting cell cycle–related genes in HCC cells (24) and recruited a partner protein, POGZ, to participate in homologous recombination DNA damage repair, thereby enhancing the survival of human osteosarcoma and cervical carcinoma cells (21). Unlike its roles in solid tumors, our study demonstrated that HRP2 acts more like a tumor suppressor in disease progression and regulates chemosensitivity to PIs in MM. In support of this assertion, analysis of the CCLE transcriptome database indicated that HRP2 expression was substantially lower in MM compared with other blood cancer subtypes and solid tumors. Using several open-access databases, we found that higher HRP2 expression predicted better survival. However, the current study did not evaluate the role of HRP2 in other hematological tumors, such as leukemia or lymphoma, which need further investigation. Of note, we also observed that KD of HRP2 in MM cells induced hyper-bone lesion activity, a phenotype that did not seem to be related to MM proliferation and which should be investigated in further studies.

In the t(4;14)-positive MM cells, highly expressed NSD2 produced a genome-wide augmentation of H3K36me2, which provided a scaffold for readers to bind with and regulate transcriptional elongation (29). Histone methylation reader proteins contribute to gene regulation and tumor pathogenesis, but the pathogenic roles of HRP2 in MM are not known. Ola Rizq et al. reported that patients with MM who have higher levels of EZH2 expression tend to respond poorly to bortezomib, thus, inhibition of both EZH1 and EZH2 sensitizes MM to PI treatment at a synergetic manner, mainly through suppression of PRC-2–dependent H3K27me3 (31). Our study showed that HRP2 levels were negatively correlated with H3K27me3 levels in MM cells. A previous study revealed that H3K36me2 and H3K36me3 inhibit the catalytic activity of PRC2, and distribution of H3K36 methylation and H3K27 methylation is mutually exclusive along chromatin (16). Our current study also revealed that the H3K36me2 reader protein HRP2 could remove H3K27me3 and *trans*-activate bortezomib-responsive genes, whose downregulation ultimately contributed to chemoresistance in HRP2^lo^ MM cells. Thus, our genome-wide data, together with the validation of specific genes, established a key role of this gene-specific regulation of HRP2 in MM cells. Our dissection of the HRP2 complex uncovered a partner, MINA, that worked as the key mediator to suppress H3K27me3 levels at the H3K36me2-abundant TSS regions of target genes. Functionally, HRP2^lo^ MM cells are speculated to have higher H3K27me3 levels and are more sensitive to inhibitors targeting H3K27me3, such as the EZH2 inhibitor tazemetostat. Our in vitro and in vivo combination administration of tazemetostat and bortezomib strongly supports this hypothesis. A previous study reported that EZH2 is highly expressed in MM and that pharmacological inhibition of EZH2 has a synergistic anti-MM effect with bortezomib (37). Although t(4;14) is widely considered a high-risk factor in the management of patients with MM and predicts a poor clinical outcome (38, 39), recent studies also suggested that an enriched gain of 1q+ in high-risk cytogenetic abnormalities leads to inferior outcomes for patients with MM (40), raising the question of whether t(4;14) alone or in combination with other factors, such as the HRP2 levels shown in our findings, determines the true high-risk status of patients with MM. Thus, this study provides insights into the underlying mechanism of resistance to PIs and, we believe, will contribute to the optimization of treatment strategies according to HRP2 levels in high-risk patients with MM.

In summary, this study underscores what we believe to be a previously unknown role of HRP2 in regulating sensitivity to PIs in MM cells and identifies an epigenetically regulated machinery based on a t(4;14) cytogenetic background. We believe the evidence from our study will accelerate efforts to identify novel therapeutics that can optimize CR rates in patients with newly diagnosed MM and provides a compelling rationale for exploring new strategies to upregulate HRP2 expression or target H3K27me3 to restore PI sensitivity in patients with RR MM.

## Methods

### Patients’ samples.

The preparation of CD138^+^ cells from patients with MM and healthy controls has been described in a previous study (28).

### Cell lines, cell viability, and flow cytometric assays.

The MM cell lines MM.1S were from the National Infrastructure of Cell Line Resource (Beijing, China); the LP-1 cells were a gift of Robert Orlowski (University of Texas MD Anderson Cancer Center, Houston, Texas, USA; and the ^WHSC1(+/+)^KMS-11 and ^WHSC1(+/–)^KMS-11 and parental lines were obtained from Horizon Discovery Ltd. The induction of bortezomib-resistant cells has been described in a previous publication (27). Cells were short tandem repeat (STR) authenticated and substantiated as mycoplasma free. CCK8 assays to detect cell proliferation and flow cytometric analysis to detect apoptosis were performed as previously described (28, 41).

### Screening of genes using the CRISPR/Cas9 sgRNA system.

The human GeCKOv2A CRISPR-KO pooled library was used to identify genes responsible for bortezomib resistance and sensitivity in LP-1 cells. First, we infected LP-1 cells with the pooled GeCKOv2 human lentivirus library at a low MOI with the vector control. After selection with 2 μg/mL puromycin, at least 8 million transduced cells were injected into the flanks of mice. After 2 weeks when tumor sizes were palpable (~5 mm diameter), 0.5 or 5 mg/kg bortezomib was administrated i.p. with DMSO as a vehicle control 3 times per week, and tumor volumes were monitored every 3 days. After treatment, at least 3 samples from each group were collected for genomic DNA extraction to ensure over 400× coverage of the GeCKO v2A library. Genomic DNA from tumors was extracted using the FastPure Cell/Tissue DNA Isolation Mini Kit (Vazyme). The sgRNA cassettes were amplified using NEBNext High-Fidelity 2× PCR Master Mix, and next-generation sequencing (NGS) was performed on an Illumina HiSeq to determine sgRNA abundance.

### Flow cytometric and cell viability assays.

Annexin V–FITC (FITC-conjugated annexin V, eBioscience) was used to label apoptotic cells. Dead cells were labeled with propidium iodide (PI) (eBioscience). The staining experiment was performed according to the product instructions. Briefly, 1 million cells were washed in cold PBS and suspended in 0.5 mL staining binding buffer. Annexin V–FITC (5 μL) and PI (1 μL), respectively, were added to the cell suspension. Cells were incubated for 15 minutes at room temperature and subjected to flow cytometric analysis. The results were analyzed using FlowJo software. For Cell Counting Kit-8 (CCK-8, APExBIO Technology) assays, MM cells were seeded at 1 × 10^5^ cells/well in triplicate in 96-well plates and incubated at 37°C in 5 % CO_2_. After incubation for 48 hours, the CKK8 reagent was added to each well and incubated for 2 hours prior to reading absorbance at 450 nm. The following formula was used to calculate cell viability: percentage = OD value of the treatment group/OD value of the control group × 100.

### Real-time qPCR.

Total RNA was extracted with the assistance of TRizol reagent (Takara), and reverse transcribed using the SuperScript III RT kit and oligo dT primers (Invitrogen, Thermo Fisher Scientific). PCR primers were purchased from Integrated DNA Technologies. Real-time qPCR was performed using iTaq Universal SYBR Green Supermix (Bio-Rad). Fold changes were calculated using the ΔΔCt method and GAPDH message as a reference. The primers used in this study are listed in the supplemental materials.

### Western blotting and co-IP.

The immunoblotting protocol was described in our previous study (42). All antibodies, venders, and dilutions are provided in the supplemental materials. The representative Western blot images from at least 3 independent experiments shown in the figures were cropped and autocontrasted. Quantification of Western blot band intensities was determined using ImageJ 1.46r software (NIH) for all samples in each group. For co-IP assays, total proteins from HRP2-overexpressing HEK293T cells were extracted with IP lysis buffer. Flag antibody or control IgG was added and vertically rotated with cell lysate overnight at 4°C. Then, protein A/G magnetic beads were added to the protein-antibody complex and incubated overnight at 4°C. The beads were washed 3 times with IP lysis buffer. The pulled-down proteins were extracted and detected by Western blotting as described above.

### Immunofluorescence staining.

Cells (1 × 10^5^) were spun down on glass slides, washed, and then fixed with 4% paraformaldehyde solution (Affymetrix) for 15 minutes at room temperature. After permeabilization and blocking, the cells were exposed to NSD2 and HRP-2 antibodies overnight at 4°C. The cells were incubated with secondary antibodies coupled to Alexa Fluor 488 goat anti–rabbit IgG (H+L) or Alexa Fluor 594 goat anti–mouse IgG (H+L) for 1 hour at room temperature in the dark. Nuclei were counterstained with DAPI. Fluorescence was visualized on a fluorescence microscope (FV-1000, Olympus).

### Mass spectrometry.

Cells were resuspended in NP-40 lysis buffer (150 mM sodium chloride, 1% NP-40, 50 mM Tris, pH 8.0) with a protease inhibitor cocktail (Roche) and centrifuged at 12,000*g* for 20 minutes at 4°C. The supernatants were incubated with anti-Flag M2 affinity gel at 4°C overnight. After washing 4 times with NP-40 lysis buffer, the Flag protein complex was eluted with Flag peptide (MilliporeSigma). The eluates were resolved on NuPAGE 4%–12% Bis-Tris gels (Invitrogen, Thermo Fisher Scientific) and stained with a silver staining kit (Pierce, Thermo Fisher Scientific). The protein bands were cut out and analyzed by liquid chromatography–tandem mass spectrometry (LC-MS/MS).

### Histone extraction.

For acid extraction of core histones, cells pellets were harvested and sequentially extracted with perchloric acid and HCl, followed by precipitation of core histones with trichloroacetic acid. Histone pellets were sequentially washed with 100% aceton/0.006% HCl and 100% aceton, dried, and resuspended for protein concentration measurement using the Bradford assay, and then loaded onto precast SDS-PAGE gels for immunoblotting.

### ChIP and ChIP-Seq.

ChIP experiments for histone modifications, HRP2, and MINA analyses were performed as described previously using antibodies against H3K27me3, HRP2, MINA, and rabbit/mouse IgG as a negative control. ChIP experiments were performed with the following modification: after cross-linking, cells were washed and resuspended in nuclei lysis buffer with protease inhibitors for 10 minutes, centrifuged, and then washed and resuspended in ChIP lysis buffer for sonication. Chromatin samples were immunoprecipitated overnight with the antibody or isotype control and 20 μL fully suspended protein A/G magnetic beads.

After washing sequentially with low-salt buffer, high-salt buffer, LiCl wash buffer, and Tris-EDTA (TE) buffer, the immunocomplexes were eluted from beads and digested with proteinase K for 2 hours at 65°C. Next, DNA was purified by spin columns and eluted in 50 μL diethylpyrocarbonate-water (DEPC-water). Input and ChIP material (10 ng) was processed using an Illumina kit (IP-102-1001). Libraries were loaded onto a HiSeq 2000 or GAIIx (both from Illumina) at 6 pM and subjected to 50 and 36 sequencing cycles, respectively. FastQC (version 0.11.8) and Cutadapt (version 2.0; https://cutadapt.readthedocs.io/en/stable/) were applied to raw sequencing reads from ChIP and input libraries for quality control and preprocessing to get the clean reads, respectively. The reads were mapped to the *Homo sapiens* genome (hg38) by Bowtie2 (version 2.2.6; http://bowtie-bio.sourceforge.net/bowtie2/index.shtml). The duplicated reads were removed using SAMtools (version 1.9; http://www.htslib.org/). Then, the peak-finding algorithm from MACS2 (version 2.1.1; https://hbctraining.github.io/Intro-to-ChIPseq/lessons/05_peak_calling_macs.html) was used to detect regions with significant enrichment of ChIP signals. The ENCODE (Encyclopedia Of DNA Elements) Processing Pipeline (https://www.encodeproject.org/pipelines/) for transcription factor and histone modification ChIP-Seq was referred to set the parameters of read mapping and peak calling. To associate the peaks with nearby genes and genomic regions, annotatePeaks.pl from HOMER (version 4.10; http://homer.ucsd.edu/homer/ngs/annotation.html) was used. The deepTools suite (version 3.2.0; https://deeptools.readthedocs.io/en/develop/) was used to produce the binding profiles and heatmaps.

### RNA-Seq and analysis.

Total RNA from cells was isolated using TRIzol (Invitrogen, Thermo Fisher Scientific) following the procedures described previously (42). Libraries were constructed and sequenced on the BGISEQ-500 platform (BGI Group). The raw sequencing reads were checked using FastQC (version 0.11.8; https://www.bioinformatics.babraham.ac.uk/projects/fastqc/). Cutadapt (version 2.0) was used for adapter trimming and low-quality filtering to get the clean data. The genome sequence and gene annotation of *Homo sapiens* (hg38, GRCh38) from GENCODE (https://www.gencodegenes.org/pages/gencode.html) were used. The gene expression was quantified by featureCounts (version 1.6.0; https://rnnh.github.io/bioinfo-notebook/docs/featureCounts.html). Based on the quantification, DEGs between sample groups were generated by DESeq2 (R, version 3.3.2; https://bioconductor.org/packages/release/bioc/html/DESeq2.html). DEGs were filtered using the cutoff of a fold change of 1.5 or greater and an adjusted *P* value of 0.05 or less.

### Drug synergy calculations.

In brief, bortezomib and tazemetostate were added to LP-1 cells, and the IC_50_ of each drug was determined. Serial dilutions were made across the IC_50_ dose range, with the IC_50_ set at 1X, and dilutions were made relative to this value. The agents were then added simultaneously for 24 hours, and CCK8 assays were performed. Data were analyzed using CalcuSyn software (Biosoft). Combination indices (CIs) were calculated, and values below 1.0 were considered to indicate synergy.

### CDX and PDX models.

Four- to 6-week-old female NOD.*Cg-Prkdc^scid^Il2rg^tm1Wjl^*/SzJ mice (Sibeifu Biology Technology) were used to establish the xenograft (*n =* 12) and intra-bone injection models (*n =* 8), as previously reported (41, 43). For the PDX model, unsorted bone marrow mononuclear cells containing 0.5 × 10^6^ to approximately 1 × 10^6^ CD138^+^ cells from patients with relapsed MM after a bortezomib-based treatment regimen were implanted intratibially into NSG mice (*n =* 6 mice/per patient). In order to monitor engraftment, recipient mice were bled weekly after inoculation, and their blood was assessed using Ig ELISA Kits (Thermo Fisher Scientific). Bortezomib alone or in combination with tazemetostat was administered twice a week for 3 weeks from the second week after inoculation, and the human CD138^+^ cells from mouse bone marrow were analyzed by flow cytometry when the treatments were terminated.

### Data availability.

The RNA-Seq and ChIP-Seq data can be publically accessed in the NCBI’s Gene Expression Omnibus (GEO) database (GEO GSE166527).

### Statistics.

Data are shown as the mean ± SD for at least 3 independent experiments. Differences between groups were determined using a paired, 2-tailed Student’s *t* test or 2-way ANOVA. Comparison of Kaplan-Meier survival curves was performed by log-rank (Mantel-Cox) test, a Pearson’s correlation test was used to determine the correlations between gene expression levels, and survival analysis and a log-rank test were done using GraphPad Prism (GraphPad Software). A *P* value of less than 0.05 was considered statistically significant.

### Study approval.

Animal studies were approved by the Committee on Animal Research and Ethics of Tianjin Medical University, and all protocols conformed to Declaration of Helsinki principles and to the Guidelines for Ethical Conduct in the Care and Use of Nonhuman Animals in Research of the World Medical Association. Signed informed consent was obtained from each individual prior to participation in the study.

## Author contributions

Jingjing Wang and ZL wrote the manuscript. Jingjing Wang, LD, YX, HJ, JG, Yixuan Wang, ZP, and MW performed experiments. XZ, QW, and LY analyzed the bioinformatics data. XL, YX, and SW were in charge of the animal studies. QL, Yafei Wang, and Jingya Wang provided the patients’ samples and clinical statistics. ZL and LZ approved the final version of this manuscript.

## Supplementary Material

Supplemental data

## Version 1 02/15/2022

Electronic publication
