# Supplementary material for: Epigenomic reprogramming via HRP2-MINA dictates response to proteasome inhibitors in multiple myeloma with t(4;14) translocation
Source: J Clin Invest. 2022 Feb 15;132(4):e149526. doi: 10.1172/JCI149526 (PMC8843744; doi:10.1172/JCI149526)
Supplement: Supplemental data [file jci-132-149526-s127.pdf]

Supplementary Figures

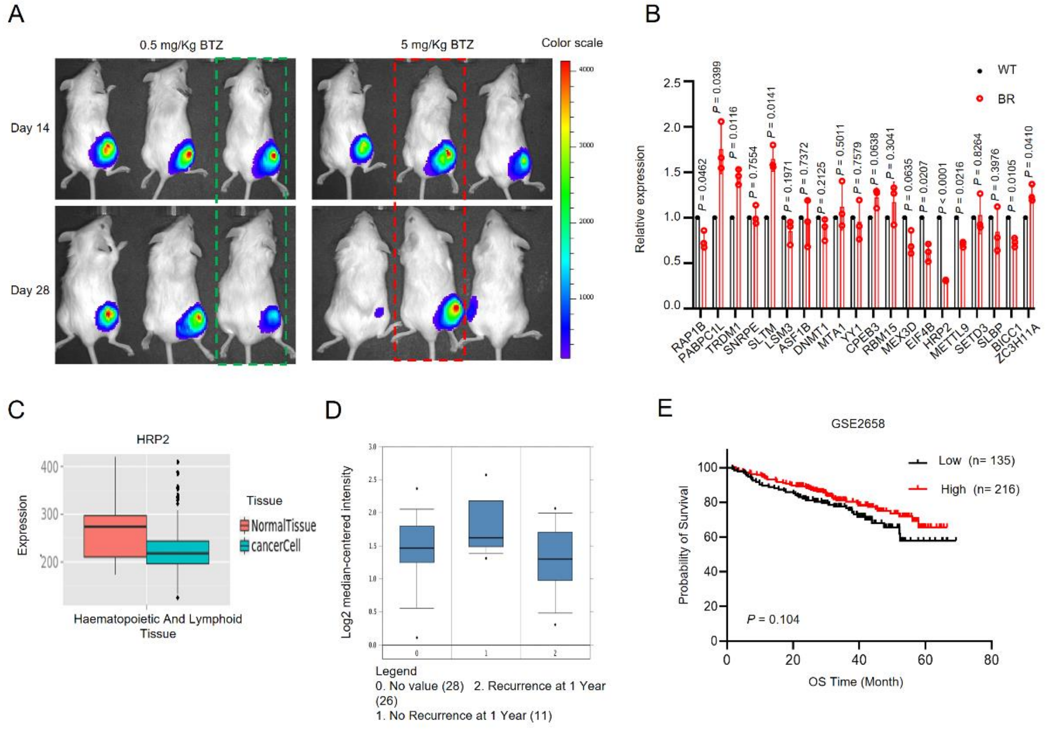

Figure S1. HRP2 is negatively correlated with MM recurrence.

(A) Representative images of intra-bone xenograft model for negative and positive selection. 1×10^6 Luciferase-LP-1 cells were injected into the femur bone marrow of NSG mice for 2 weeks, and then treated with 0.5mg/kg or 5mg/kg bortezomib (i.p.) for another 2 weeks. Green frame, representative tumor for negative selection; red frame, representative tumor for positive selection (n = 3). **(B)** mRNA expression levels of the top 20 enriched genes from the screening results in WT and BR LP-1 cells (n = 3). Two-sided *P* value determined by Student's *t* test; mean ± SD. **(C)** HRP2 mRNA in normal tissue and cancer cell of Haematopoietic and Lymphoid tissue from the Broad Institute Cancer Cell Line Encyclopedia database. **(D)** HRP2 expression and recurrence status of MM patients after one-year treatment from Oncomine database. **(E)** Correlation of *HRP2* mRNA expression with overall survival (OS) in myeloma patients from Zhan's database (n = 351, TT2). Cutoff= 491.10, the threshold of HRP2 signal. *P* values were determined by Pearson Coefficient and Log-ranks test.

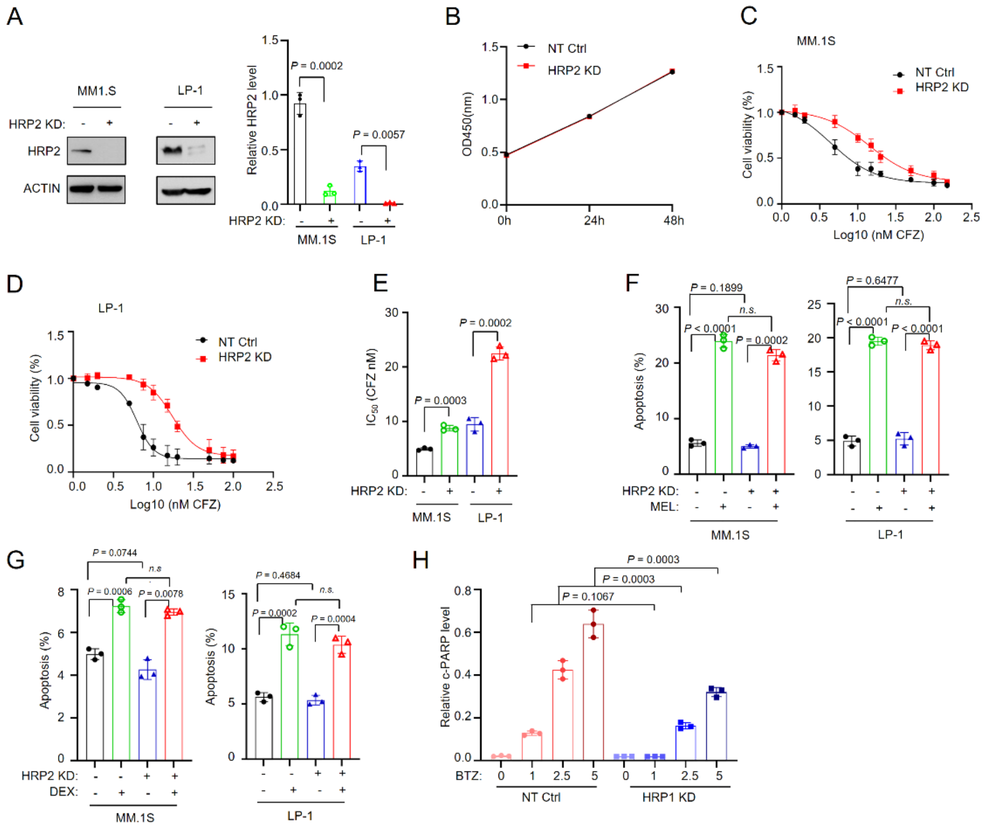

Figure S2. Genetic ablation of HRP2 promotes myeloma cell drug resistance.

(A) Efficacy of HRP2 knockdown (KD) by lentiviral-based shRNA in MM.1S and LP-1 cells (n = 3). **(B)** Cell proliferation of LP-1 cells infected with non-target control (NT Ctrl) or shRNA targeting HRP2 (HRP2 KD) (n = 3). **(C)** and **(D)** Alteration of sensitivity to carfilzomib (CFZ) treatment in the NT Ctrl or HRP2 KD MM.1S and LP-1 cells (n = 3). **(E)** Comparison of the IC_{50} of CFZ in the NT Ctrl or HRP2 KD MM.1S and LP-1 cells (n = 3). Two-sided *P* values were determined by Student's *t* test; mean ± SD. **(F)** Flow cytometry assay shows percentage of apoptosis in the NT Ctrl or HRP2 KD MM.1S and LP-1 cells upon Melphalan (Mel) treatment for 48 h (n = 3). **(G)** Flow cytometry assay shows percentage of apoptosis in the NT Ctrl or HRP2 KD MM.1S and LP-1 cells upon Dexamethasone (Dex) treatment for 48 h. Two-sided *P* values were determined by Student's *t* test; mean ± SD of 3 independent experiments. **(H)** Quantitative analysis of cleaved PARP for figure 2G.

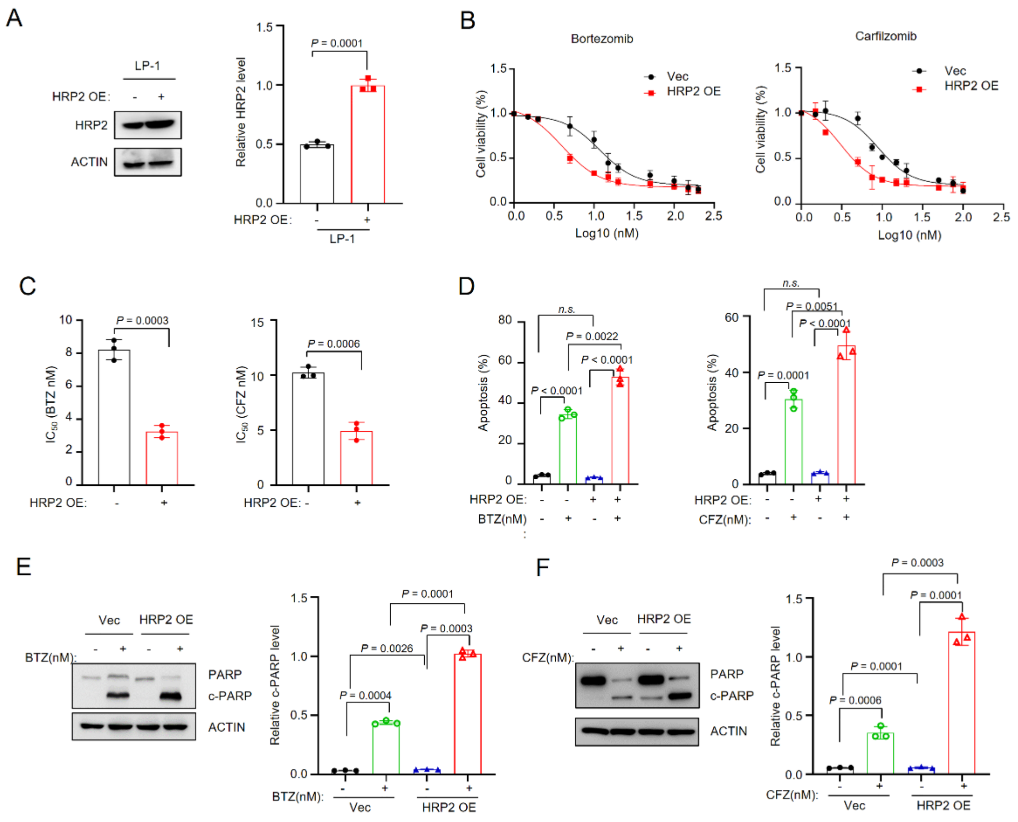

Figure S3. Overexpression of HRP2 enhances sensitivity to PIs in MM cells.

(A) Western blot and quantification analysis of HRP2 protein level in LP-1 cells infected with lentivirus carrying HRP2-overexpressing vector (n = 3). **(B)** Alteration of sensitivity to BTZ (5nM) and CFZ (5nM) treatment in LP-1 cells infected with vector (Vec) or HRP2 overexpressing plasmid (HRP2 OE) (n = 3). **(C)** Comparison of the IC_{50} of BTZ and CFZ in Vec or HRP2 OE LP-1 cells (n = 3). Two-sided *P* value was determined by Student's *t* test; mean ± SD of 3 independent experiments. **(D)** Flow cytometry analysis shows the apoptosis percentage of Vec and HRP2 OE LP-1 cells upon BTZ (5nM) or CFZ (5nM) treatment for 48 h (n = 3). Two-sided *P* was calculated using Student's *t* test; mean ± SD of 3 independent experiments. Western blot showing the cleaved PARP and the quantification analysis in the Vec or HRP2 OE LP-1 cells treated with **(E)** BTZ (5nM) for 48h (n = 3), and **(F)** treated with CFZ (5nM) for 48 h (n = 3).

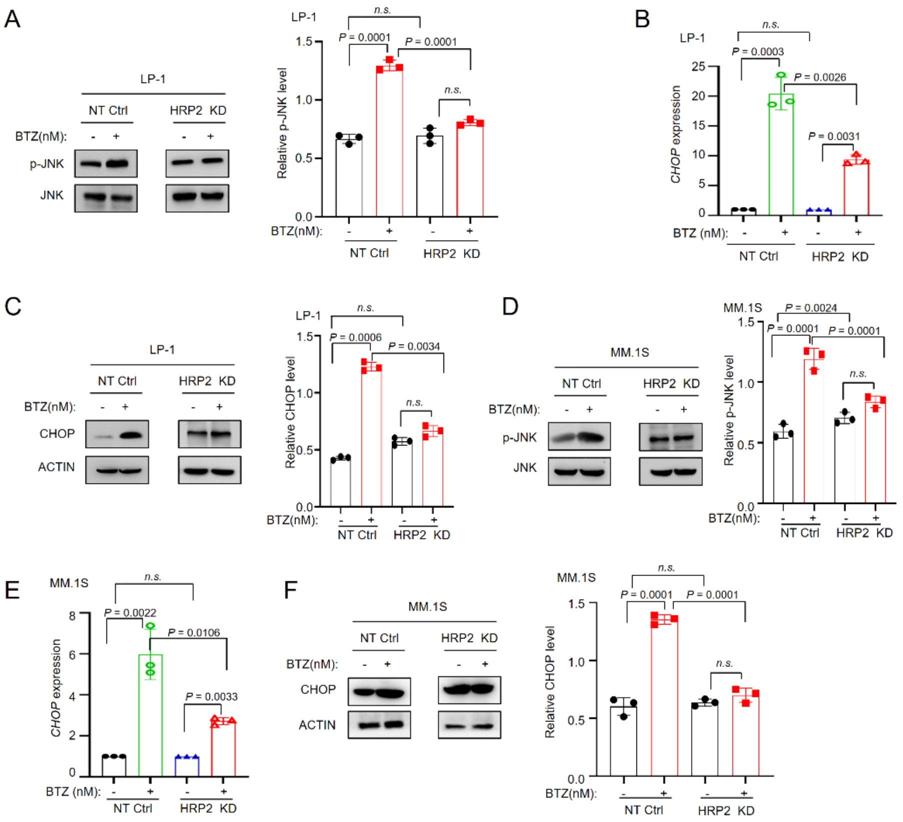

Figure S4. Knockdown of HRP2 inactivates stress-response pathway

(A) Western blot showing the levels of phosphorylated JNK (p-JNK) and the quantification analysis in the NT Ctrl or HRP2 KD LP-1 cells (n = 3). **(B)** *CHOP* mRNA levels in the NT Ctrl or HRP2 KD LP-1 cells treated with DMSO or BTZ (5nM) for 48 h (n = 3). Two-sided *P* value was determined by Student's *t* test; mean ± SD of 3 independent experiments. **(C)** Western blot showing the CHOP level and the quantification analysis in the NT Ctrl or HRP2-KD LP-1 cells upon BTZ (5nM) treatment for 48 h (n = 3). **(D)** Western blot showing the levels of phosphorylated JNK (p-JNK) and the quantification analysis, **(E)** mRNA and **(F)** protein level of CHOP and the quantification analysis in the NT Ctrl or HRP2 KD MM.1S cells treated with DMSO or BTZ (5nM) for 48 h (n = 3). Two-sided *P* calculated using Student's *t* test; mean ± SD of 3 independent experiments.

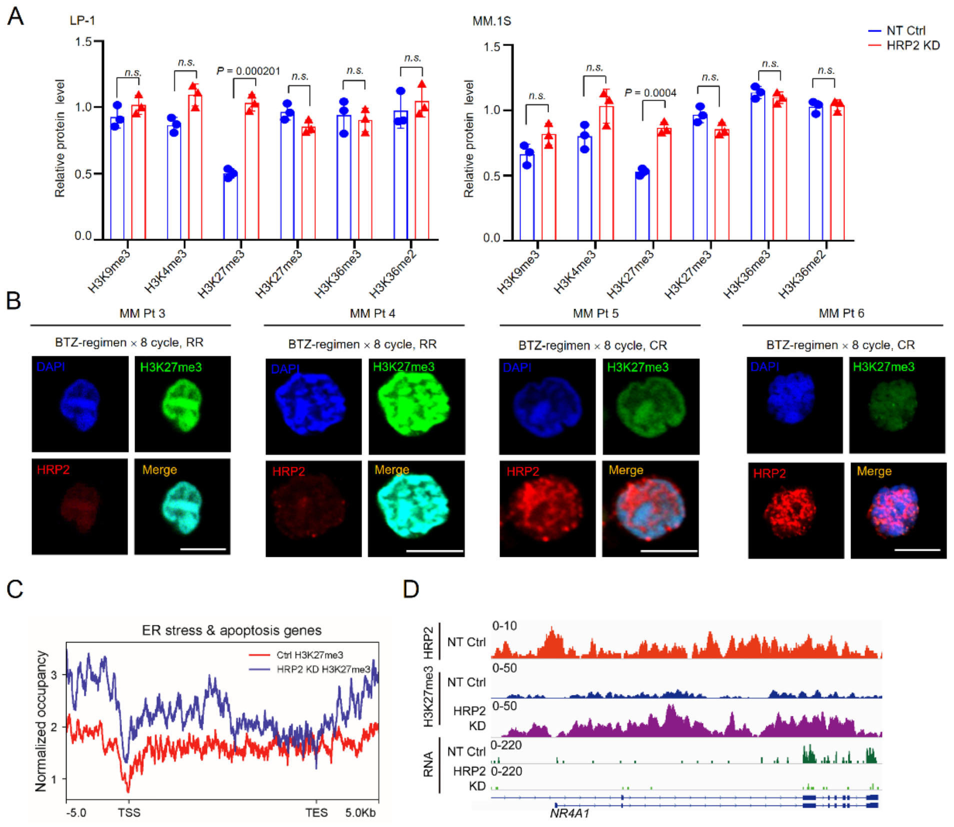

Figure S5. Suppression of HRP2 desensitizes MM cells to chemotherapeutics.

(**A**) Quantitative analysis of Western blotting in figure 5A (n = 3). **(B)** Confocal fluorescence images of HRP2 (red) and H3K27me3 (green) in two $CD138^{+}$ plasma cells from MM patients with disease progression and two patients acquired CR (scale bars: 10 μm; n = 3). **(C)** Tag density profile of H3K27me3 distribution on endoplasmic reticulum (ER) stress and apoptosis related genes in LP-1 cells. **(D)** Gene tracks shows representative ChIP-Seq profiles for the indicated proteins and histone marks at the *NR4A1* gene loci.

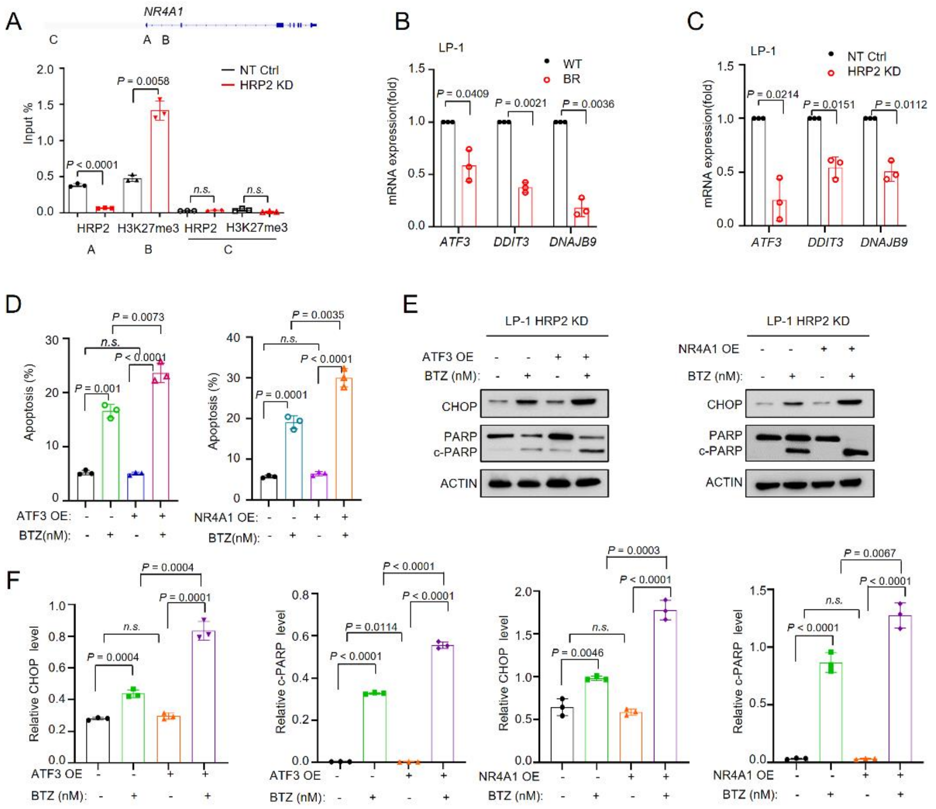

Figure S6. ATF3 and NR4A1 expression associates with resistance to bortezomib in HRP2 KD MM cells.

(A) ChIP-qPCR of H3K27me3 and HRP2 at *NR4A1* gene loci in HRP2 KD LP-1 cells (n = 3). PCR primers were designed according to ChIP-seq peaks of corresponding proteins on these gene loci. Schematic representation of PCR primer design is provided. Two-sided *P* value determined by Student's *t* test; mean ± SD of 3 independent experiments. **(B)** qPCR shows mRNA expressions of *ATF3, DDIT3,* and *DNAJB9* in wild type (WT) or BTZ-resistant (BR) LP-1 cells (n = 3). **(C)** qPCR shows mRNA expressions of *ATF3, DDIT3,* and *DNAJB9* in the NT Ctrl or HRP2 KD LP-1 cells (n = 3). Two-sided *P* calculated using Student's *t* test; mean ± SD of 3 independent experiments. **(D)** Flow cytometry assay shows apoptosis percentage in HRP2 KD LP-1 cells with or without ATF3 and NR4A1 overexpression (OE) upon BTZ (5nM) treatment for 48 h (n = 3). Two-sided *P* was calculated using Student's *t* test; mean ± SD of 3 independent experiments. **(E)** Western blotting and **(F)** quantitative analysis of CHOP and cleaved PARP after ATF3 or NR4A1 OE in HRP2 KD LP-1 cells treated with BTZ (5nM) for 48 h (n = 3).

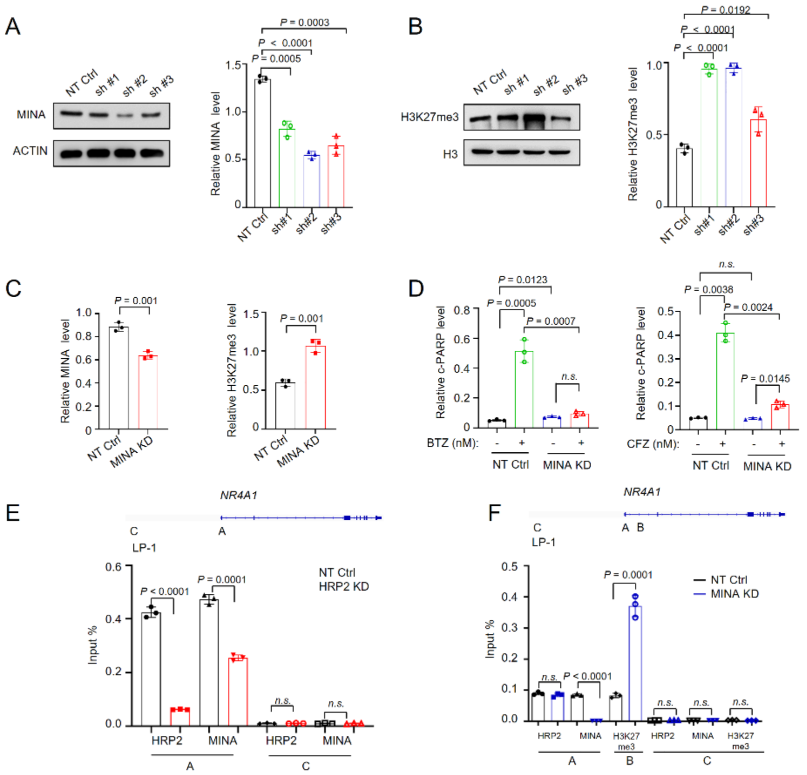

Figure S7. Knockdown of MINA interferes drug resistant gene expression.

(A) Western blot showing the efficacy of three shRNAs targeting MINA expression and the quantification analysis in LP-1 cells after lentiviral-carrying infection for 72 h (n = 3). **(B)** Western blot showing levels of H3K27me3 and the quantification analysis in LP-1 cells with MINA knockdown by 3 shRNAs for 72 h (n = 3). **(C)** Quantitative analysis the Western Blot of MINA and H3K27me3 levels in LP-1 cells with MINA knockdown by lentivirus-carrying shRNA (n = 3). **(D)** Quantitative analysis the Western Blot of cleavage of PARP in the NT Ctrl and MINA KD LP-1 cells treated with bortezomib (BTZ, 5 nM) for 48 h (n = 3). **(E)** ChIP-qPCR of HRP2 and MINA at *NR4A1* gene loci in HRP2 KD LP-1 cells (n = 3). PCR primers were designed according to ChIP-seq peaks of corresponding proteins on these gene loci. Schematic representation of PCR primer design is provided. *P* value determined by Student's *t* test for 3 independent experiments. **(F)** ChIP-qPCR of HRP2, MINA and H3K27me3 at *NR4A1* gene loci in MINA KD LP-1 cells (n = 3). PCR primers were designed according to ChIP-seq peaks of corresponding proteins on these gene loci. Schematic representation of PCR primer design is provided. Two-sided *P* value determined by Student's *t* test; mean ± SD of 3 independent experiments.

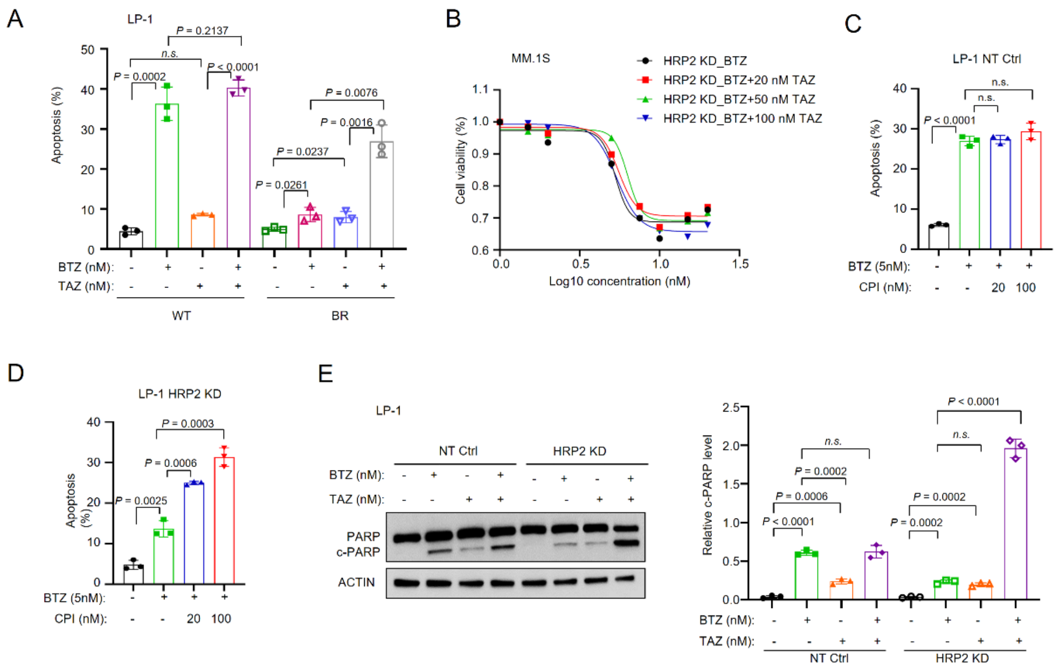

Figure S8. Combination of H3K27me3 inhibitor with bortezomib overcomes chemoresistance in HRP2-low MM cells.

(A) Apoptosis of the wild type (WT) or bortezomib-resistant (BR) LP-1 cells treated with BTZ (5 nM) in presence or absence of TAZ (100 nM) for 48 h (n = 3). Two-sided *P* value determined by Student's *t* test; mean ± SD of 3 independent experiments. **(B)** Cell viability of the HRP2 KD MM.1S cells treated with 5 nM bortezomib (BTZ) and various concentrations of tazemetostat (TAZ) for 48 h. Apoptosis of the NT Ctrl **(C)** or HRP2 KD **(D)** LP-1 cells treated with BTZ (5 nM) in presence or absence of two dosages of CPI-169 (CPI) for 48 h (n = 3). Two-sided *P* value determined by Student's *t* test; mean ± SD of 3 independent experiments. **(E)** Western blot showing the cleaved PARP and the quantification analysis in the NT Ctrl or HRP2 KD LP-1 cells treated with BTZ (5nM) with or without TAZ (20nM) for 48 h (n = 3).

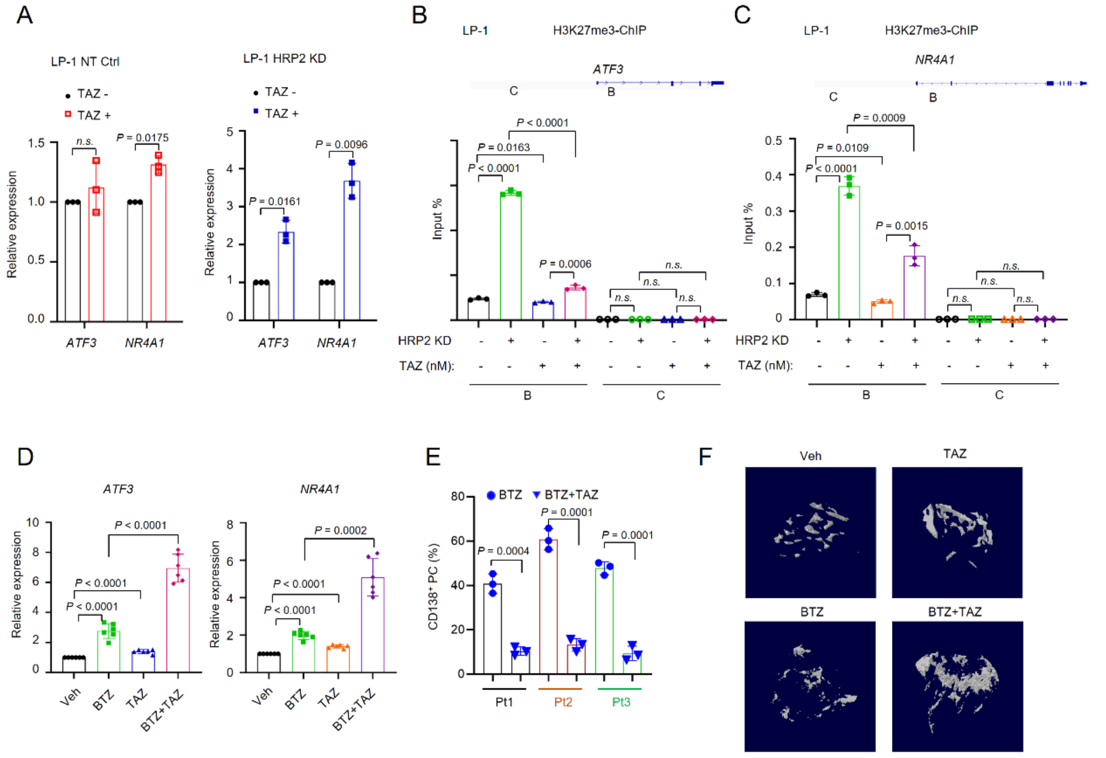

Figure S9. Inhibition of H3K27me3 sensitizes resistant MM cells to bortezomib therapy.

(A) *ATF3* and *NR4A1* mRNA levels in NT Ctrl or HRP2 KD LP-1 cells treated with TAZ (20nM) for 48 h (n = 3). Two-sided *P* value determined by Student's *t* test; mean ± SD of 3 independent experiments. **(B)** ChIP-qPCR of H3K27me3 at *ATF3* and **(C)** at *NR4A1* gene loci in the NT Ctrl or HRP2 KD LP-1 cells in presence or absence of TAZ (20nM) for 48 h (n = 3). PCR primers were designed according to ChIP-seq peaks of corresponding proteins on these gene loci. Schematic representation of PCR primer design is provided. Two-sided *P* value determined by Student's *t* test; mean ± SD of 3 independent experiments. **(D)** mRNA expression of *ATF3* and *NR4A1* in tissues from mice bearing xenografts and treated with vehicle (Veh), bortezomib (BTZ), tazemetostat (TAZ), or the combination of BTZ and TAZ (BTZ, 1 mg/Kg, i.p.; TAZ, 0.5mg/Kg, p.o.; n = 6 mice/group). Two-sided *P* value determined by Student's *t* test; mean ± SD of 3 independent experiments. **(E)** Percentage of human CD138^{+} cells in the bone marrow of NSG mice after receiving 3 weeks of BTZ or BTZ combined with TAZ treatment. n = 6 mice/patient sample, n = 3 mice/group. Two-sided *P* value

determined by Student's *t* test; mean ± SD of 3 independent experiments. **(F)** Representative 3D reconstructions of bone trabecula in metaphyseal regions bearing BR LP-1 cells for the femur in the figure 7H (n = 6 mice/group).

Differentially expressed genes in LP-1 cells upon HRP2 knockdown

Up-regulated genes	Down-regulated genes
IGHG1	MKX
AK4	NR4A1
HFE	CDKN1A
CXCL10	MEI1
PFKFB4	CARD11
HSPA8P7	TRIB1
CAVIN4	CLIP2
HSPA8	GNAO1
HSPA6	TRIB3
AL606807.1	KCNK12
MT2A	ZBP1
BNIP3	WNK4
HSPH1	NOS1AP
ZNF823	SLC37A1
LDLRAD3	LRP1
HILPDA	NXPH4
CHORDC1	C1QTNF6
IGFBP3	MST1
OLFML2A	ADGRA2
FUT11	HDGFL2
ALMS1-IT1	SV2C
HSPE1P2	LY9
GPD2	NAV1
GLUL	FOSL2
BHLHE40	DNAJB9
FKBP4	ITGA3
LGMNP1	DUSP4
LYAR	DAB2IP
DNAJA1	TBC1D9
CRYBB1	RAB31
PFKFB3	PPDPF
HSPE1	OSGIN1
AP001033.2	NELL2
PUS7	CEBPB
FAM162A	H1FX-AS1
DNAJB1	H6PD
MT1X	FGFR3
HSP90AA1	LINC01136
MRPS6	SPRED2
LHFPL2	UBALD2

ZNF230	ISG20
LDHAP3	CHPF
ZNF416	ETV5
ZFP82	ETV1
HSP90AA2P	CDT1
HS3ST3B1	CD28
MSMO1	PTPRVP
TSKU	IZUMO4
AC083843.3	JMJD8
SLC5A3	GTPBP2
LGMN	TNFRSF10B
PIK3AP1	WFS1
P4HA1	PPM1M
WDR77	RNF150
AHSA1	INAVA
DGAT2	MED16
POLR3G	PLSCR1
TNFRSF1B	RAB26
ANKRD27	IL21R
NOP16	TMEM44-AS1
PRDM10	CDC42EP3
PDGFC	SLC1A4
SPIRE1	TUBGCP6
WIPF3	MAP1B
IGLC5	DNASE2
SLC6A6	FAM167A
GBE1	TECPR1
SPR	TP53INP1
TIMM8A	B4GALT3
SLC25A30	MYO1D
LDHAP7	CASKIN2
MACC1	PYCR1
ID1	BMP8A
EMP2	ZNF688
HES6	XYLT1
TSPOAP1	SYVN1
GPATCH4	WIPI1
CA2	GPR160
ESF1	GAA
DDX10	KLF9
MTCH2	HIP1
TMEM200A	ABCA3
BHLHE22	BCL2

SLIRP	CCR10
SELENOW	ZNF629
MALAT1	FAT1
PSTK	AKNA
FAM53B	C22orf46
MPHOSPH6	CEP68
GEMIN5	PRSS16
TRAF5	KIF13B
BCL6B	SMOX
HSPA4L	ITGB7
PDSS1	SBF1
ADGRA3	TNFRSF13B
TUBD1	PHYKPL
FABP5	RNPEPL1
SERINC5	CUL9
DDN	PNKP
ZNF566	RASA2
STAMBPL1	SUN2
SQLE	GMPPB
FAM210A	MYL5
SLC19A2	PAQR6
FGFR2	ABCD1
IDI1	TKFC
ZNF567	MAP3K12
DNAJC2	SLC25A45
FAM117B	FAM234A
BEND4	P2RY6
POU3F2	TFEB
ADAT2	PTK2B
BAG2	TES
CGN	TUFT1
NLN	CFLAR
SORD	TMEM175
SSBP1	MELTF
SCARB1	PDCD4
ZNF615	PPCDC
HSPB1	CHST12
INTS13	BMP6
FAM49B	SH2B2
PDCD2L	IRF4
INTS8	GALNT17
CAMK4	EIF2A
PNPT1	ARID5A

MSANTD3	KLF6
FTX	H1FX
SNRPA1	PKMYT1
KCTD6	LRATD2
ZNF780A	TCEA2
CAV2	MUS81
PAK1IP1	CIC
POLR3K	PRKD2
RUSC1-AS1	P4HB
ZNF480	ATF3
BRI3BP	ERN1
MIR17HG	DDIT3
BLVRA	AC138655.1
MYB	PLXDC2
ZNF530	AC233296.1
PPP2R1B	AL512306.2
ZNF700	PKDREJ
NFKBIE	AC136297.1
GRPEL1	TLCD3B
HMGCR	TMEM9B-AS1
MICOS10	FAM43A
SLC25A17	AC107959.3
JPT1	TMEM198
SLC25A13	C15orf65
HMGCS1	THORLNC
BCL11A	FAM171B
EBNA1BP2	AKAP3
PON2	PLEK
PGK1	AL139220.2
GPD1L	FNBP1P1
TTC27	LINC01547
TPI1	AC016727.1
PYGL	ROBO3
RRP9	NLRP1
PNO1	RNF122
ENO1	AC112484.1
TIMM21	AC092117.1
ACAT2	AC007240.3
SFT2D2	CDNF
GTF2E2	AL356599.1
POLR1B	AC091729.3
METTL8	KCNA2
E2F5	PLXNA2

SNX27	INAFM1
ARHGEF3	AC139530.1
RPIA	KCNA3
ADK	IRS2
EXOSC7	APLP1
BRIX1	TTLL1
MARCKSL1	BDNF-AS
RUVBL1	ETV4
NUP35	OSER1-DT
TRIM58	HLCS
RLF	AC099343.3
GALNT18	PPP3CB-AS1
FASTKD1	AC046134.2
POP1	SPATA20
KNSTRN	GALNT3
LSM6	CFAP44
ZNF143	ATP6AP1L
ENAH	ABTB1
ABCC4	FAT4
MRPL17	PGAP3
CTSC	ZNRF2P1
BAG4	FKBP10
MARS2	TUBE1
CSTB	SLC16A14
TPST1	FICD
CHST2	AC087741.1
NAA25	MXD3
MTATP6P1	SIDT1
BCCIP	NLRX1
EIF5B	SH3RF1
PARP12	JUND
LPCAT4	HVCN1
VCL	DNHD1
NOL6	VDAC2P3
ZNF134	C5
SLC5A6	AC006252.1
SLC29A1	EBF4
PTMAP3	DAPK1
TIMM23	SLFN5
SLC41A1	AC008014.1
IGF2BP2	ECE1
LYN	APBB2
HPRT1	FYN

MRPS12	SCRN1
HNRNPA2B1	RND3
LCP1	TMEM8B
MLKL	TRPM8
CCT5	C1orf226
GLRX5	WDR25
CACYBP	AC064807.1
LINS1	HSPA13
B4GALT5	SLC24A1
MAK16	TMEM267
TBC1D4	NKIRAS1
NSDHL	ARHGEF40
KDM1A	NRGN
SSB	PLA2G6
PPIL1	C21orf58
RFK	LTB4R
PRPF3	NCAM1
PRDX1	AC007207.1
NOL11	C1RL
KIAA1549L	TSPYL2
RPF2	WDR45
RARG	ZNF367
ITGB8	TMEM147-AS1
RARS	PLD3
PGAM1	PCDH9
TENT4B	AGA
MRPL50	EIF4E3
UBE2N	AL365361.1
CPNE3	SEC24D
GOLGA8A	PRIMPOL
STIP1	SELENON
HIST1H4I	HIVEP2
DNAAF5	TRAM2-AS1
MRTO4	SYNE1
FAM136A	TRIM11
ALDOC	YIPF2
HSP90AB3P	NR1D2
NSUN2	RECQL5
HIST1H2BK	FBXL16
CTPS1	RRNAD1
RBM25	CUTALP
CDCA7	TRIOBP
HSP90AB1	SREBF1

RBM47	STX5
TMEM138	C16orf58
HEATR1	ALDH6A1
SRSF2	DOCK11
GTF3A	ZBED3
CHKA	IRF1
SLC4A7	E2F2
NUP93	ADGRL1
HOMER2	AC016747.1
WDR3	CC2D1A
DYNLL1	BCL2L1
ERO1A	HDAC6
GABPB1	BLVRB
MRPL15	CYB5D2
SEH1L	PAN2
MRPS25	CCDC82
PPARGC1B	GABPB1-AS1
FDFT1	HYOU1
SSBP2	SELPLG
ID2	PTCH1
GNPDA1	AIG1
CCDC86	INPP4A
GEMIN6	LMBRD2
COA4	SLC7A1
HSD17B7	EEF2
	NQO2
	PIGV
	RNF157
	IFI35
	AC090136.3
	BRCA1
	PLAAT4
	CD99L2
	C19orf48
	LRP10
	CASP10
	CDK2AP2
	NRBP2
	SEL1L
	TCEA1P2
	ATXN2L
	FECH

Key resources of this study

Category	Source	Cat. No.
**Antibodies**		
Anti- HDGF2 Polyclonal antibody	proteintech	15134-1-AP
Anti- Phospho-SAPK/JNK (Thr183/Tyr185) Antibody	Cell signaling technology	9215S
Anti-CHOP (D46F1) Rabbit mAb	Cell signaling technology	5554S
Anti-Di-Methyl-Histone H3 (Lys27) (D18C8) XP® Rabbit mAb	Cell signaling technology	9728S
Anti- Tri-Methyl-Histone H3 (Lys27) (C36B11) Rabbit mAb	Cell signaling technology	9733S
Anti- Tri-Methyl-Histone H3 (Lys4) (C42D8) Rabbit mAb	Cell signaling technology	9751S
Anti- Tri-Methyl-Histone H3 (Lys9) (D4W1U) Rabbit mAb	Cell signaling technology	13969S
Anti-Histone H3 (di methyl K36) antibody-ChIP Grade	Abcam	ab9049
Anti Tri- Methyl-Histone H3 (Lys36) (D5A7) XP® Rabbit mAb	Cell signaling technology	4909S
MINA53 Monoclonal Antibody (M532)	Thermo Fisher	39-7300
Anti-PARP	Cell signaling technology	9532
Anti-Histone H3 antibody - Nuclear Loading Control and ChIP Grade	Abcam	ab1791
Anti-rabbit β-actin	Abclonal	AC006
Goat Anti-Rabbit IgG-HRP	Sigma-Aldrich	A0545
ANTI-FLAG® M2-Peroxidase	Sigma-Aldrich	A8592
Rabbit Anti Mouse IgG-HRP	Sigma-Aldrich	A9044-2ML
Anti-rabbit IgG	Proteintech	30000-0-AP
Anti-mouse IgG	Proteintech	B900620
**Chemicals, Peptides and Recombinant Proteins**		
3×FLAG peptide	Sigma-Aldrich	F4799
FLAG Peptide	Sigma-Aldrich	F3290
**Drugs**		
Bortezomib (PS-341)	SelleckChem	S1013
Carfilzomib(PR-171)	SelleckChem	S2853
Puromycin 2HCL	SelleckChem	S7417
Melphalan, minimum 95%	Sigma-Aldrich	M2011
Dexamethasone	Sigma-Aldrich	D4902
CPI-169	SelleckChem	S7616
Tazemetostat (EPZ-6438)	SelleckChem	S7128
**Enzymes**		

RNase A, DNase and protease-free	Thermo Fisher	EN0531
Proteinase K Solution, ChIP grade	Thermo Fisher	26160
Benzonase Nuclease	Sigma-Aldrich	E1014-25KU
Multiscribe Reverse Transcriptase	ABI	4308228
dNTP mix	ABI	362275
**Plasmids**		
pLenti-CMV-HDGFL2-3*Flag	Public Protein/Plasmid Library	PPL01991-4b
MINA/MINA53 cDNA ORF Clone, Human, C-DYKDDDDK (Flag®) tag	Sino Biological	HG14082-CF
pCMV3-WHSC1-Flag	Sino Biological	HG11530-CF
pITA-ATF3-Flag	Self-construction	
pITA-NR4A1-Flag	Self-construction	
HDGFRP2-shRNA1	Shanghai genechem	SHCLNV-NM_032631
HDGFRP2-shRNA2	Shanghai genechem	SHCLNV-NM_032631
HDGFRP2-shRNA3	Shanghai genechem	SHCLNV-NM_032631
$PSPAX_2$	Gift from Dr. Xudong Wu, Tianjin Medical University, Dept. Cell Biology	
PMD_2G	Gift from Dr. Xudong Wu, Tianjin Medical University, Dept. Cell Biology	
**Critical Commercial Assays**		
EvaGreen 2X qPCR MasterMix	ABI	MasterMix-R
5×All-In-One RT MasterMix	abm	G490
Luciferase assay Kit	Promega	E1910
Duolink™ In Situ Detection Reagents Red	Sigma-Aldrich	DUO92008
Pierce BCA Protein Assay Kit	Thermo SCIENTIFIC	23225
AxyPrep DNA Extraction Kit	AXYGEN	295 AP-GX-250G
AxyPrep Plasmid Miniprep Kit	AXYGEN	183 AP-MN-P-250G
Plasmid Maxi Kit(25)	QIAGEN	12163
EnVision G12 Doublestain System,Rabbit/Mouse(DAB+/Permanent Red)	Dako	K5361
SuperSignal West Dura Extended Duration Substrate	ThermoFisher	34580
9002 SimpleCHIP® Kit	Cell Signaling	22188S
Simple CHIP® Kits-20C-Reagents	Cell Signaling	45061S
ChIP-grade Protein A/G Magnetic Beads	Thermo SCIENTIFIC	26162
ANTI-FLAG M2 Affinity Gel	Sigma-Aldrich	A2220
CellTiter 96 Aqueous One Solution	Promega	G358B
NuPAGE 4-12% Bis-Tris Gel	Invitrogen	NP0335BOX
Phosphatase Inhibitor Cocktail(100×)	Cell Signaling	587OS

Annexin V-FITC Apoptosis Kit	Sigma-Aldrich	APOAF-50TST
Pierce® Protein G Plus Agarose	Thermo Scientific	22852
Human CD20 MicroBeads	Miltenyi Biotec	MB17-R0829
Human CD138 MicroBeads	Miltenyi Biotec	130-105-961
Whole Blood Column Kit	Miltenyi Biotec	MB17-R0189
DeadEnd™ Fluorometric TUNEL System	Promaga	G3250
Ficoll-Paque PLUS endotoxin tested	GE Healthcare	17-1440-02
LS Columns(25 columns)	Miltenyi Biotec	130-042-401
TRIzol Reagent	Ambion, Life Science	15596018
Opti-MEM®I(1×) Reduced Serum	Gibco, Life Technologies	31985-070
Opti-protein XL Marker	ABM	G266
PageRuler Prestained protein Ladder	ThermoFisher Scientific	26616
1Kb Ladder DNA Marker	Biomed	MD114
1Kb DNA Ladder	TIANCEN	MD111
100bp DNA Ladder	TRANS	BM301
BM15000 DNA Marker	Biomed	MD106
1Kb Plus DNA Ladder	Solarbio	M1500
PEI-Transferrinfection Kit	ThermoFisher Scientific	BMS1003
IgA Human ELISA Kit	ThermoFisher Scientific	BMS2096
IgG Human ELISA Kit	ThermoFisher Scientific	BMS2091

Sequences of all primers used in this study

Primers for q-PCR

**Primers sequence**	
Human GAPDH-F	TTGCCCTCAACGACCACTTT
Human GAPDH-R	TGGTCCAGGGGTCTTACTCC
Human HRP2-F	CGTGAAGAGGTGCCTGAATG
Human HRP2-R	ACTTCTGCTGCCTTCTCCAT
Human CHOP-F	TGAAAGGAAAGTGGCACAGC
Human CHOP-R	TGGTGCAGATTCACCATTCG
Human ATF3-F	AGCCATTGGAGAGCTGTCTT
Human ATF3-R	AATGGCCAGTGTGTTAAGGC
Human DNAJB9-F	AGAGCGCCAAATCAAGAAGG
Human DNAJB9-R	TCAGCATCCGGGCTCTTATT
Human RAP1B-F	GTGACAGCGTGAGAGGTTCG
Human RAP1B-R	ATTGCTGTAAATTGCTCCGTTCC
Human PABPC1L-F	GCTGAATGACCGCAAAGTCT
Human PABPC1L-R	CCCAAACTGGGAGAAGAGGT
Human TRDM1-F	TGACACTGACGTGAACCAGT
Human TRDM1-R	AAGCACACGGACCTTCTACA
Human SNRPE-F	GCGGGTGTGCTCTTTGTGAA
Human SNRPE-R	TGAAGATGAGGTTGATGGGCT
Human SLTM-F	AGACAGGCGAGAAGTACGAG
Human SLTM-R	CTGGTGGGTCTGGAAGGATT
Human LSM3-F	CGACGTAGACCAGCAACAAA
Human LSM3-R	ACAGTTTCTTCCACATCTCCCA
Human ASF1B-F	CTCGGCTGAGAGTGAGGAAT
Human ASF1B-R	CAGGTGATGAGGACCACAGT
Human DNMT1-F	CAGCACAAACTGACCTGCTT
Human DNMT1-R	AGCCAGTGATCCACCATTCA
Human MTA1-F	TTGATGCCCAGTAGGGGTCT
Human MTA1-R	GGTAGGACTTCCCGTTGAGC
Human YY1-F	GGGATAACTCGGCCATGAGA
Human YY1-R	TTCGAACGTGCACTGAAAGG
Human CPEB3-F	CCTTCGAGCTGTTGAACTGG
Human CPEB3-R	TTCAGCTCTGGGTCCGTATC
Human RBM15-F	TCCCAGCAGTTCCTGGATTC
Human RBM15-R	AAACCAAACCCACGGACAAT
Human MEX3D-F	GAAGGAGGACGTGGAGATGG
Human MEX3D-R	CACCTGGATGGTGGTCTGTC
Human EIF4B-F	TGGCCATCAAATTGCGCTTA
Human EIF4B-R	TTCCTCCATCTGTTGGCGAT

Human METTL9-F	CACCAGTGGTATGTGTGCAA
Human METTL9-R	GCCCGATTTCTCAATGCTGT
Human SETD3-F	TGGCCATCAAATTGCGCTTA
Human SETD3-R	TTCCTCCATCTGTTGGCGAT
Human SLBP-F	TCCCAAGACACCTTCGACAA
Human SLBP-R	AGCCACCTTCCAGAGTTTGA
Human BICC1-F	GGACAACCAGCAGGAGTAGA
Human BICC1-R	ACATCAGCACCAAAGGAAGC
Human ZC3H11A-F	CTCAGCGTCGGAGTCAAGAG
Human ZC3H11A-R	GCAGGGAATATCCCCAAATCCTA
Human HRP2-shRNA-1	CCGGAGAAGAACACAGACGTGGTGGCTCGAGC CACCACGTCTGTGTTCTTCTTTTTTTG
Human HRP2-shRNA-2	CCGGAGTACGGGAAGCCCAACAAGACTCGAGT CTTGTTGGGCTTCCCGTACTTTTTTTG
Human HRP2-shRNA-3	CCGGGTTTGCCCTAAAGGTCGACAGCTCGAGC TGTCGACCTTTAGGGCAAACTTTTTTG
Human MINA-shRNA-1	CCGGGCAACGATTCAGTTTCACCAACTCGAGTT GGTGAAACTGAATCGTTGCTTTTTG
Human MINA-shRNA-2	CCGGCAAGTTTCCTTACCTGCGTATCTCGAGAT ACGCAGGTAAGGAAACTTGTTTTTTG
Human MINA-shRNA-3	CCGGGACCTGAAACTTACTACAGATCTCGAGA TCTGTAGTAAGTTTCAGGTCTTTTTTG

Primers for ChIP q-PCR

**Primers sequence**	
ATF3-A-F	TGAAGGCAACCCCGAGAAAA
ATF3-A-R	GTGAGAGTCCCAGGTGTGTG
ATF3-B-F	GCAAAGATGGGATGGGGTGA
ATF3-B-R	AAGAAGGCTCCCATCACTGC
ATF3-C-F	TGGGACTGACAGAGGTCCAG
ATF3-C-R	AGTGTTGCTTCAGGAACATTGTA
NR4A1-A-F	GAAACCTTCCAAGTGCCTGC
NR4A1-A-R	TAGTTCTCCCTGCTCTCCCC
NR4A1-B-F	CCAGGGCTGGGGACTTTTAG
NR4A1-B-R	AGTAAGTGTGCGTGGAAGGG
NR4A1-C-F	CCAAGGGAGTGAACCCCATC
NR4A1-C-R	CAACCCCAGTCCATGAGCTT
